# Altered intestinal microbiota enhances adenoid hypertrophy by disrupting the immune balance

**DOI:** 10.3389/fimmu.2023.1277351

**Published:** 2023-11-28

**Authors:** Wenxin Liu, Huier Jiang, Xiling Liu, Yue Zheng, Yanan Liu, Fen Pan, Fangyuan Yu, Zhi Li, Meizhen Gu, Qingqing Du, Xiaoyan Li, Hong Zhang, Dingding Han

**Affiliations:** ^1^Department of Clinical Laboratory, Shanghai Children’s Hospital, School of Medicine, Shanghai Jiao Tong University, Shanghai, China; ^2^Institute of Pediatric Infection, Immunity, and Critical Care Medicine, School of Medicine, Shanghai Jiao Tong University, Shanghai, China; ^3^Shanghai Key Laboratory of Forensic Medicine, Shanghai Forensic Service Platform, Academy of Forensic Science, Ministry of Justice, Shanghai, China; ^4^Department of Pathology, Shanghai Children’s Hospital, School of Medicine, Shanghai Jiao Tong University, Shanghai, China; ^5^Department of Otolaryngology-Head and Neck Surgery, Shanghai Children’s Hospital, School of Medicine, Shanghai Jiao Tong University, Shanghai, China; ^6^Medical School, Guangxi University, Nanning, China

**Keywords:** gut microbiota, adenoid hypertrophy, immune response, fecal microbiome transplantation, children

## Abstract

**Introduction:**

Adenoid hypertrophy (AH) is a common upper respiratory disorder in children. Disturbances of gut microbiota have been implicated in AH. However, the interplay of alteration of gut microbiome and enlarged adenoids remains elusive.

**Methods:**

119 AH children and 100 healthy controls were recruited, and microbiome profiling of fecal samples in participants was performed using 16S rRNA gene sequencing. Fecal microbiome transplantation (FMT) was conducted to verify the effects of gut microbiota on immune response in mice.

**Results:**

In AH individuals, only a slight decrease of diversity in bacterial community was found, while significant changes of microbial composition were observed between these two groups. Compared with HCs, decreased abundances of *Akkermansia*, *Oscillospiraceae* and *Eubacterium coprostanoligenes* genera and increased abundances of *Bacteroides*, *Faecalibacterium*, *Ruminococcus gnavus* genera were revealed in AH patients. The abundance of *Bacteroides* remained stable with age in AH children. Notably, a microbial marker panel of 8 OTUs were identified, which discriminated AH from HC individuals with an area under the curve (AUC) of 0.9851 in the discovery set, and verified in the geographically different validation set, achieving an AUC of 0.9782. Furthermore, transfer of mice with fecal microbiota from AH patients dramatically reduced the proportion of Treg subsets within peripheral blood and nasal-associated lymphoid tissue (NALT) and promoted the expansion of Th2 cells in NALT.

**Conclusion:**

These findings highlight the effect of the altered gut microbiota in the AH pathogenesis.

## 1 Introduction

The adenoid is a component of Waldeyer’s ring, located at the back of the pharyngeal vault, and often extends to the upper border of the posterior pharyngeal wall. The adenoid enlarges physiologically in early childhood until about the age of six years, and then shrink from eight-ten years of age (1). Nasal-associated lymphoid tissue (NALT) in rodents, a paired lymphoid cell aggregate, was considered to be the equivalent of the Waldeyer’s ring of humans (2). Similar to the characteristic of adenoid physiological growth, NALT was present at birth and the overall size diminished during aging (3). Under normal physiological conditions, enlargement of the adenoid may regress when the pathological stimuli are discharged. However, the pathological conditions remain constant in many cases after treatment (4). The space between adenoid and palatine becomes narrowed or even closed, which leads to nasal obstruction, acute otitis media, otitis media effusion, mouth breathing, nocturnal snoring, and obstructive sleep apnea syndrome (5). If not treated promptly and effectively, these symptoms can cause serious comorbidities, such as adenoid face, obesity, cardiovascular disease, and cognitive impairment (6).

At present, the prevalence of adenoid hypertrophy in children was estimated to be as high as 34% (7). The most common diagnostic methods for adenoid hypertrophy are rhinoscopy and nasopharyngeal endoscopy (8). However, children with adenoid hypertrophy have a lower acceptability of invasive diagnosis. The first step of hypertrophy intervention usually focuses on addressing the underlying causes. Surgical removal of enlarged tissues is usually required for AH patients with persistent obstruction. Many children undergo adenoidectomy annually. Although surgery is more effective than drug therapy (9), the procedure carries a risk of excessive bleeding and infection (10). Studies of adenotonsillectomy outcomes have reported that complete postoperative normalization of the apnea/hypopnea index (AHI) was observed in only 25% of the patients postoperatively (11). A worsening indication over time was observed in 68% of the cases (12). Moreover, the adenoids can regrow following adenotonsillectomy. Thus, patients may undergo additional revision adenoidectomies at a rate of approximately 13.3% (13)–14.5% (14).

At the present stage, the gut microbiome has been recognized as the “second genome” or “an organ” of the human body, which communicates and interplays with other organs or systems locally and distantly in various ways, such as metabolites and immune response (15). About 70% of the lymphoid immune cells in our body are distributed in gut associated lymphoid tissue (GALT) (16). After exposure to the microbe, the dendritic cells within GALT recognize the antigens. They either induce an inflammatory response by activating T helper lymphocytes, or an anti-inflammatory response via regulatory T cells. Therefore, the gut microbiome plays a crucial role in shaping the immune system during the first year of life and in the modulation of host immunity and the progression of immune-mediated diseases later (17). It has been pointed that dysbiosis of gut microbiota are closely connected with the immunological dysregulation of the respiratory tract diseases and their responses to treatment. For example, children at three months of age, who carry significantly decreased *Lachnospira*, *Veillonella*, *Faecalibacterium*, and *Rothia* genera are at high risk of asthma (18). While children of three months old carrying an increase in the relative abundance of *Streptococcus* and *Bacteroides* species and decrease in *Bifidobacterium* species and *Ruminococcus gnavus* are prone to develop atopic asthma at age five years (19). In addition, the low abundance of *Bifidobacteria*, *Akkermansia*, and *Faecalibacterium* genera are also associated with atopy and asthma (20). The allergic airway inflammation influenced by gut microbiota is mediated by short-chain fatty acids (SCFAs) that induce the expression of FOXP3, and boost the level of T regulatory cells (Tregs) and the production of IL-10 (21). Previous studies indicated that the progression of allergic rhinitis (AR), a common inflammation condition at upper respiratory tract, is also related with gut microbe, such as *Prevotella* and *Escherichia* (22). The diversity of gut microbiota and the abundance of *Firmicutes* phylum in patients with allergic rhinitis is lower, while the relative abundance of *Bacteroidetes* is higher. To date, an increasing number of probiotics have shown beneficial effects in the prevention of allergic airway inflammation in mouse models (23, 24) and humans (25).

Recent studies have suggested that AH may be due to an imbalance in the immune status. Children with genetic variants in Toll-like receptors (TLRs), which are important regulators of the immune response, have an increased risk for AH (26). The ratio of peripheral Th17/Treg cells and the expression level of cytokines such as IL-17, IL-10, and TGF-β in children with adenoid hypertrophy are significantly correlated with the increased blockage degree (27). Moreover, the dysregulation of IL-4 and IL-5 expression and Treg levels are observed in children with adenoid regrowth after adenotonsillectomy (28). A similar diet plays an important role in asthma (29) and allergic rhinitis (22), food allergy is also significantly associated with the onset of adenoid hypertrophy (30). In patients with AH, milk was the most prevalent sensitizing allergen (31). Furthermore, the positive rate of food intolerance is higher in adenoidal hypertrophy cases, with eggs, milk, and cod as the top three allergens (32). Taken together, the gut microbiota may be involved in the development of AH. Currently, studies pay close attention to the effects of local bacteria on the adenoid in children with adenoid hypertrophy. However, little is known about the relationship between gut microbiome and adenoid hypertrophy in pediatric patients.

In this study, we performed 16S ribosomal RNA (rRNA) gene sequencing of 219 fecal samples from patients with adenoid hypertrophy and healthy controls to characterize the signatures of the gut microbiome and their functional potential. Based on the differences in the gut microbiota abundance, a biomarker panel discriminating between AH and HC was identified. We further validated the diagnostic and predictive potential of the panel as a noninvasive tool for AH using the discovery and validation sets, respectively. In addition, to investigate the causal effects of gut microbiota on adenoid hypertrophy, stool samples from pediatric patients with AH and healthy controls were intragastrically transplanted into mice to study the immune response in the peripheral blood and NALT.

## 2 Materials and methods

### 2.1 Subject recruitment

All participants provided written informed consent, and fecal samples were collected from the Department of Clinical Laboratory, Shanghai Children’s Hospital, School of Medicine, Shanghai Jiao Tong University. The current gold standard for the diagnosis of AH is nasendoscopy (NE) (33). Here, all the patients with AH were undergone NE examination and with an blockage ratio over 75%. Exclusion criteria for AH patients were as follows: a) development of other underlying diseases, including cancers, gastrointestinal diseases, and inflammatory diseases; b) antibiotics, probiotics, or prebiotics used in the past four weeks; c) surgical treatment experienced within the past three months; and d) presence of missing clinical information. The HCs were physically examined and found to have no rhinitis, snoring, or adenotonsillar hypertrophy. Healthy controls met the exclusion criteria.

### 2.2 Fecal sample collection and DNA extraction

Fresh fecal samples from all participants and mice were collected from the Department of Clinical Laboratory, Shanghai Children’s Hospital, School of Medicine, Shanghai Jiao Tong University, and stored at -80°C as soon as possible (<30 min). Genomic DNA was extracted using Quick-DNA Fecal/Soil Microbe Kits (Zymo Research, USA), according to the manufacturer’s instructions. DNA concentration was measured by a Qubit 2.0 (Invitrogen, USA). The DNA quality was estimated using a Bioanalyzer 2100 (Agilent, USA).

### 2.3 PCR amplification, sequencing, and sequence process

The extracted DNA samples were amplified using a set of primers, 338F (5’-ACTCCTACGGGAGGCAGCAG-3’) and 806R (5’-GGACTACHVGGGTWTCTAAT-3’) (34) targeting the high-variant V3-V4 region of the 16S rRNA gene via PCR. The PCR products were detected on a 2% w/v agarose gel, and the targeted bands were extracted and purified using an AxyPrep DNA Gel Extraction Kit (Axygen, USA). High-throughput paired-end sequencing was performed on an Illumina MiSeq instrument according to the manufacturer’s instructions by Shanghai Mobio Biomedical Technology Co., Ltd., China. Raw data were deposited in the NCBI Sequence Read Archive database (Accession Number: PRJNA893900). All paired-end sequenced reads were merged using FLASH version 1.2.11 (35) with default parameters. Customed criteria were used to filter and assign overlapped reads into different samples: a) no ambiguous bases (N) allowed in reads; b) the maximum mismatch rate in the overlapping region over 0.05 not allowed; and c) no mismatch in library primers allowed. Chimeric sequences were removed using UCHIME version 4.2.40 (36), and the 16S “golden standard” from the Broad Institute (version microbiome util-r20110519, http://drive5.com/uchime/gold.fa) was used as a reference to match the OTUs.

### 2.4 Bacterial diversity and taxonomic analysis

Randomly selected reads from all samples were clustered into OTUs through the UPARSE pipeline (37) with an identity threshold of 0.97. The sequences were annotated with the RDP classifier version 2.6 (38) and set the confidence level as 0.5 according to the developer’s documents (http://rdp.cme.msu.edu/classifier/class_help.jsp#conf). Based on OTUs profiles analysis, bacterial α-diversity was determined by the species richness indices (the Chao 1 estimator and the Ace estimator) and species diversity indices (the Shannon index and the Simpson index), using the R program package “vegan.” The rarefaction curves and species accumulation curves were used to determine the saturation of sample numbers and sequencing depth. The R program package “vegan” (http://www.R-project.org/) also was used to calculate the microbiome distance between samples based on the OTUs profile, which was statistically tested by the PERMANOVA test.

Differences in bacterial taxonomy, including phylum, class, order, family, and genus, between both groups were analyzed using the Wilcoxon rank-sum test. The association between clinical parameters and taxa was estimated using Spearman’s correlation coefficient. The LEfSe method (http://huttenhower.sph.harvard.edu/lefse/) was applied to screen out communities with significant differences in AHs. Significance was detected using the Kruskal-Wallis (KW) sum-rank test, and the effect size of each feature was evaluated using the LDA score. PICRUSt 2 (http://github.com/picrust/picrust2) was used to predict the gut microbe-related KEGG module profiles and pathways (39).

### 2.5 Microbial markers for AH and random forest classification models

Random Forest was used to select discriminative OTUs for the classifier model using OTU profiles of the discovery set with significant differences. The generalization error was obtained using five-fold cross validation. The minimum number of OTUs with the smallest cross-validation error and SD at the corresponding point was chosen. The POD index was predicted with the identified marker OTUs. The classification model was evaluated using the ROC curve (R 3.3.0, pROC package), and the AUC was used to represent the ROC effect.

### 2.6 Antibiotic treatment and fecal microbiota transplantation

Twenty-one female BABL/c mice (three weeks of age) were purchased from SPF (Beijing) Biotechnology Co., Ltd. Mice were administered by oral gavage with dissolved antibiotics containing ampicillin (1 g/mL), neomycin sulfate (1 g/mL), metronidazole (1 g/mL), and vancomycin (0.5 g/mL) twice daily for seven days (40). The bacterial suspension was prepared by randomly selecting ten stool samples from each group of AHs or HCs individuals and mixed at equal proportions. 48 hours after the last antibiotic administration, the mice were intragastrically administered with bacterial suspension samples or phosphate-buffered saline (PBS) as a control. An aliquot of 0.2 ml suspension was introduced by gavage into each mouse twice weekly for eight weeks.

### 2.7 Quantitative real-time PCR

Fecal pellets were collected from mice without antibiotic treatment (D0), treated with antibiotics for three days (D3), and for seven days (D7), respectively. Stool samples were weighed and stored at -80°C until processing. Total bacterial DNA was extracted using a TIANamp Stool DNA Kit (TIANGEN BIOTECH CO., LTD, Beijing, China), according to the manufacturer’s instructions. Total RNA was extracted from homogenizing NALT tissues using Direct-zol™ RNA Miniprep (Zymo Research, USA), and then reverse transcription of cDNA was performed using HiScript III All-in-one RT SuperMix Perfect (Vazyme Biotech Co., Ltd, China), according to the manufacturer’s instructions. The abundance of total bacteria was evaluated using SYBR green quantitative polymerase chain reaction with primers 515F (5’-GTGCCAGCMGCCGCGGTAA-3’) and 805R (5’-GACTACCAGGGTATCTAATCC-3’) to target 16S rRNA genes (41, 42). In addition, RT-qPCR was performed to measure the mRNA expression of TLR4 using β-actin as an internal control. The forward primers of TLR4 and β-actin were 5’-ATGGCATGGCTTACACCACC-3’ and 5’-GTGACGTTGACATCCGTAAAGA-3’, respectively. The reverse primers of TLR4 and β-actin were 5’- GAGGCCAATTTTGTCTCCACA-3’ and 5’- GCCGGACTCATCGTACTCC-3’, respectively.

### 2.8 Flow cytometry

Peripheral blood of 30 participants in each group selected randomly was collected into collection tubes containing Ethylene Diamine Tetraacetie Acid (EDTA) and immediately centrifuged at 1000 g for 10 min. Plasma was stored at -20°C. The cytokines including IL-5, IFN-α, IL-2, IL-6, IL-1β, IL-10, IFN-γ, IL-8, IL-17, IL-4, IL-12P70 and TNF-α were captured by antibody-coated beads and measured by flow cytometry following the manufacturer’s instructions (Raisecare, China).

Whole blood (50 μL) was incubated with antibodies at room temperature for 15 min. Subsequently, to eliminate the interference of RBCs, the lysing solution was incubated in the dark for 15 min. The cells were then washed three times with PBS, resuspended in 0.5 mL, and analyzed on a flow cytometer (BD FACS Canto II, Becton, Dickinson and Company, USA). For CD4/CD8 ratio detection, PerCP anti-mouse CD45 (1:200, 103129, BioLegend, San Diego, CA, USA), APC anti-mouse CD3 (1:200, 100235, BioLegend), FITC anti-mouse CD4 (1:200, 100405, BioLegend), and PE anti-mouse CD8α (1:200, 100707, BioLegend) were used. PerCP anti-mouse CD45, FITC anti-mouse CD4, APC anti-mouse CD25 (1:200, 101909, BioLegend), and PE anti-mouse CD127 (1:200,135009, BioLegend) antibodies were used to gate Treg cells.

To analyze Th17 cells, 100 μL whole blood was added to an equal volume of RPMI 1640 culture medium (GIBCO, Grand Island, NY, USA). One microliter of Phorbol 12-myristate 13 acetate (PMA)/Ionomycin and one microliter of Brefeldin A (BFA)/Monensin were added and mixed evenly, followed by incubating for four hours. After stimulation, 100 μL of the cell mixture was incubated with PerCP anti-mouse CD45 and FITC anti-mouse CD4 antibodies for 15 min. After lysing the red blood cells and washing, the cell pellets were fixed and permeabilized using the Transcription Factor Staining Buffer Kit (Multi Sciences, China). An additional PE anti-mouse IL-17A (1:200, 506903, BioLegend) antibody was added before flow cytometry analysis.

### 2.9 Histological staining

The NALT samples were fixed in a 4% paraformaldehyde solution. The fixed tissues were rinsed overnight with running water, dehydrated in an alcohol gradient, cleared in xylene, and embedded in paraffin. Sections with a thickness of 4 μm were stained with hematoxylin and eosin (HE) and sealed with neutral gum.

### 2.10 Immunohistochemistry staining

Tissue sections were dewaxed and dehydrated successively, and rinsed with PBS. Heat-mediated antigen retrieval was performed in Tris-EDTA buffer pH 9.0 for 20 min, followed by rinsing with PBS. The sections were then treated with 0.3% H_2_O_2_ for 10 min to inactivate endogenous peroxidase. The sections were then incubated separately with anti-FOXP3 antibody (1:100, ab215206, Abcam, Cambridge, MA, USA), anti-ROR gamma antibody (1:1000, ab207082, Abcam), anti-GATA3 antibody (1:500, ab199428, Abcam), and anti-T-bet/Tbx21 antibody (1:1000, ab300451, Abcam) at 37°C for one hour and rinsed with PBS. Next, the sections were incubated with goat anti-rabbit IgG secondary antibody at 37°C for 20 min, rinsed with PBS, and stained with DAB. Finally, the sections were counterstained with hematoxylin and observed under a light microscope.

### 2.11 Statistical analysis

Means and standard deviations analyzed by Student’s t-test or Mann-Whitney U test were used to represent continuous variables, including age, BMI, onset age, duration of disease, and the ratio of Th17/Treg in mice plasma. Percentages analyzed by the Chi-square test were used to represent categorical variables, including sex. Results of mice plasma were analyzed using one-way analysis of variance (ANOVA) comparing means for AH, HC, and PBS groups, or the Kruskal-Wallis test for skewed data. All statistical tests were performed using GraphPad Prism 9.0.0 (GraphPad, La Jolla, CA, USA). Statistical significance was defined as *p* < 0.05 (two tailed).

### 2.12 Ethical statement

This study was performed in accordance with the guidelines of the Declaration of Helsinki and approved by the Shanghai Children’s Hospital Ethics Review Committee (2021R124-E01).

## 3 Results

### 3.1 Clinical characteristics of the recruited participants

A total of 219 participants were recruited for this study, including 119 patients with AH and 100 healthy controls (HCs). The sex, age, and body mass index (BMI) of AH patients and healthy controls were matched (**Table 1**). There were no significant differences in these indices between the two groups (**Supplementary Table 1**). In addition, the serum levels of 12 cytokines including IL-5, IFN-α, IL-2, IL-6, IL-1β, IL-10, IFN-γ, IL-8, IL-17, IL-4, IL-12P70 and TNF-α were detected in the selected participants. Among them, the levels of IL-17 and IL12P70 were significantly changed, with an about two-fold increase for IL-17 (**Supplementary Figure 1**, **Supplementary Table 2**).

**Table 1:** Clinical and demographic characteristics of recruited participants (AH, adenoid hypertrophy; BMI, body mass index).

Clinical indices	Recruited participants (n = 219)		*P* value
	AH (n = 119)	HC (n = 100)	
Sex (Male/Female)	66/53	57/43	0.8193
Age (year, mean ± SD)	4.59 ± 1.42	4.86 ± 1.59	0.1825
BMI (kg.m^-2^, mean ± SD)	16.06 ± 2.30	16.61 ± 3.78	0.2091
Onset age (year, mean ± SD)	3.50 ± 1.27	–	–
Duration of disease (day, mean ± SD)	400.30 ± 248.30	–	–

Continuous variables were expressed as means ± standard deviation. Categorical variables were expressed as percentages. Continuous variables were compared using Student *t* test or Mann-Whitney *U* test, and categorical variables were compared using Chi-square test or Fisher’s exact test.

### 3.2 Comparison of gut microbial diversity between AH and HC

After 16S rRNA sequencing, 21001482 raw reads were obtained from all fecal samples with an average length of 456 bp. Unrepeated reads of high quality were clustered into 1781 OTUs with a similarity of 97% (**Supplementary Table 3**). The flattening trend of species accumulation curves suggested that the sample size of both groups was sufficient for downstream analysis (**Supplementary Figure 2** ). In addition, OTUs richness nearly approached saturation in both groups as the number of sequencing reads increased, as indicated by the rarefaction curve (**Supplementary Figure 3**).

**Figure 1:**
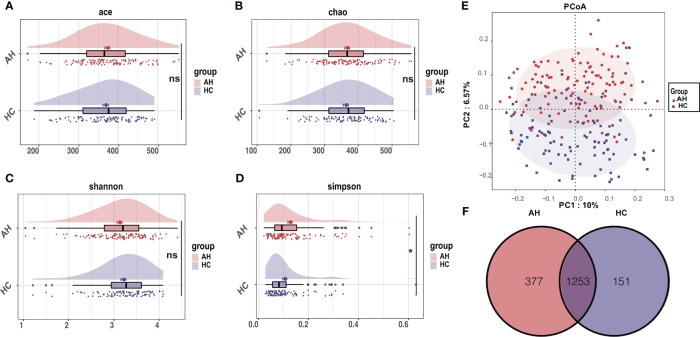
Differential characteristics of gut microbial diversity in AH patients (n = 119) and HCs (n = 100). **(A–D)** The cloudplot of α-diversity indices including the Ace index, the Chao index, the Shannon index, and the Simpson index showed differences between AH patients and HCs. **(E)** The PCoA analysis displayed the significant discrepancies in the gut microbial community between AH and HCs. (*, *P* < 0.05). **(F)** A Venn diagram showing the overlaps of OTUs between groups. AH, adenoid hypertrophy; HCs, healthy controls; OTUs, operational taxonomic units; PCoA, principal co-ordinates analysis.

Within-individual diversity of the gut microbiome is often related to health status. To assess the gut microbial α-diversity, species richness indices, including Ace and Chao, and species diversity indices, including Shannon and Simpson indices, were compared between AH patients and HCs. Only a slight decrease in α-diversity in AH patients was inferred using Simpson’s index (*p* < 0.05). There were no significant differences in the other three indices between the two groups (**Figures 1A–D**, **Supplementary Table 4**).

Principal coordinate analysis (PCoA) based on the systematic relationship of OTUs distribution was performed to compare the composition of the microbial communities of distinct samples between the different groups. The β-diversity composition of the gut microbiome was significantly different between AH and healthy controls, as inferred by permutational multivariate analysis of variance (PERMANOVA) (*p* < 0.001, **Figure 1E**). The difference in β-diversity was also revealed by non-metric multidimensional scaling analysis (NMDS) (**Supplementary** **Figure 4A**). The consistency between β-diversity and group classification based on diseases was validated by canonical analysis of principal coordinates (CAP) (p < 0.001) (**Supplementary Figure 4B**). Among the total of 1781 OTUs, 1253 OTUs were shared in both groups. Strikingly, 377 OTUs were unique to AH, whereas 151 OTUs were uniquely assigned to HCs (**Figure 1F**).

### 3.3 Alternations of gut microbiome taxonomic composition in AH patients

**Figure 2:**
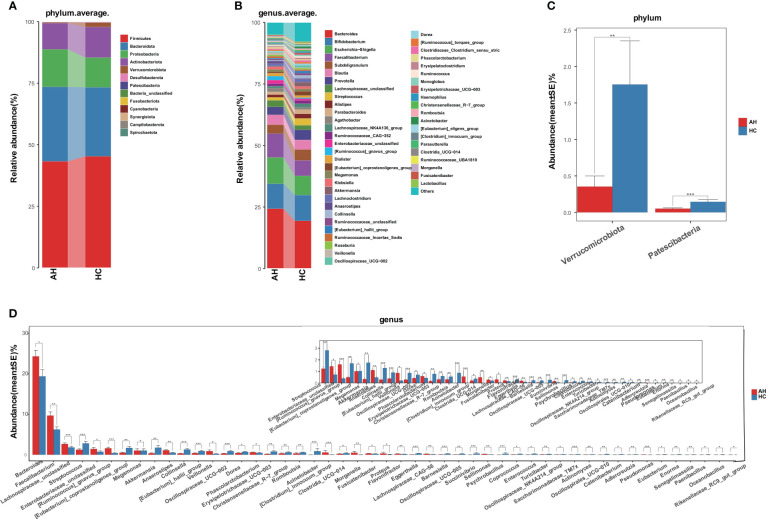
Gut microbiome taxonomic composition in AHs (n = 119) and HCs (n = 100). **(A)** Average compositions and relative abundance of the gut microbial community in both groups at the phylum level. **(B)** Average compositions and relative abundance of the gut microbial community in both groups at the genus level. **(C)** At the phylum level, the abundances of two phyla were significantly reduced in AHs. **(D)** Different relative abundance of the gut microbiota between AHs and HCs. AH, adenoid hypertrophy; HCs, healthy controls. ***, *P* < 0.001; **, *P* < 0.01; *, *P* < 0.05.

Next, we compared the gut microbial taxonomic composition in both AH and HC groups (**Figures 2A, B**, **Supplementary Tables 5**, **6**). At the phylum level, *Firmicutes*, *Bacteroidetes*, *Proteobacteria*, and *Actinobacteria* were dominant in the gut microbiome. *Bacteroides*, *Bifidobacterium*, *Escherichia-Shigella*, *Faecalibacterium*, and *Subdoligranulum* were the most abundant genera. Compared with HCs at the phylum level, *Verrucomicrobiota* and *Patescibacteria* were significantly decreased in the AH group (**Figure 2C**, **Supplementary Table 7**). Additionally, 28 genera were significantly enriched, whereas 76 genera were significantly decreased in AH. At the genus level, the relative abundances of *Bacteroides*, *Faecalibacterium*, and *Ruminococcus gnavus* were higher, while *Streptococcus*, *Eubacterium coprostanoligenes*, *Akkermansia*, and *Oscillospiraceae* were lower than those of HCs. (**Figure 2D**, **Supplementary Table 8**).

We also compared the gut microbial composition between the two groups at class, order, and family levels. At the class level, *Negativicutes* was significantly enriched, while four bacterial communities, including *Bacilli*, *Coriobacteriia*, and *Verrucomicroniae*, were reduced in AHs compared to HCs (**Supplementary Figure 5A**, **Supplementary Table 9**). Compared with HCs at the order level, *Pseudomonasales* and *Burkholderiales* were increased, and *Lactobacillales*, *Coriobacteriales*, and P*eptostreptococcales-Tissierellales* showed a significant decrease in AH (**Supplementary Figure 5B**, **Supplementary Table 10**). At the family level, seven bacterial communities, including *Veillonellaceae*, *Acidaminococcaceae*, and *Sutterellaceae*, were enriched, while 17 bacterial communities, such as *Akkermansiaceae*, *Streptococcaceae*, *Erysipelatoclostridiaceae*, and *Coriobacteriaceae* were significantly decreased in AHs (**Supplementary Figure 5C**, **Supplementary Table 11**).

### 3.4 The key discriminative microbial feature in AHs

**Figure 3:**
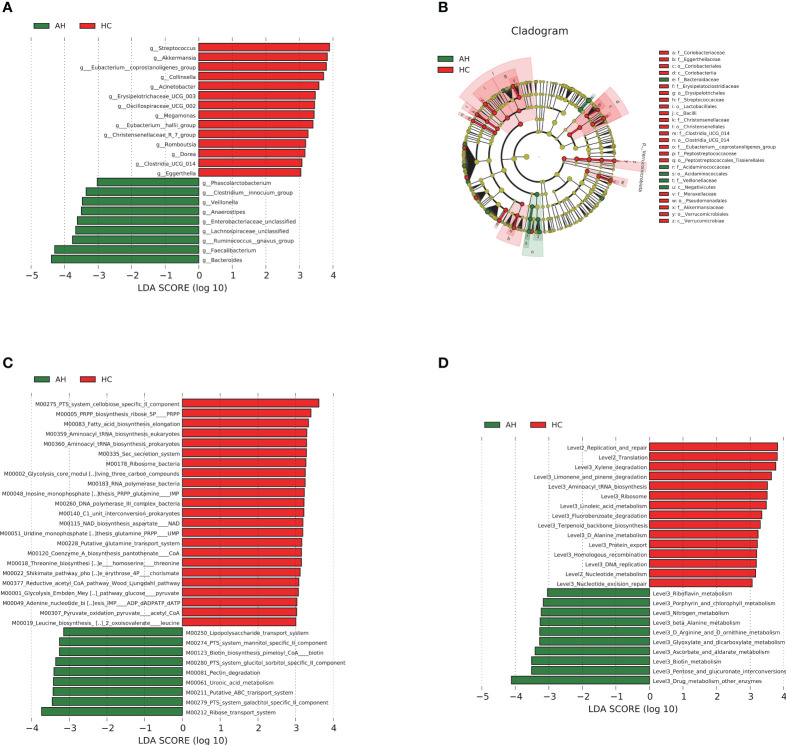
LEfSe analysis based on LDA effect size demonstrated differences in taxonomic composition of AH patients (n = 119) compared to HCs (n = 100) showed by histogram **(A)** and cladogram **(B)**. |LDA scores| > 3, *P* < 0.05. The significantly changed KEGG modules **(C)** and pathways **(D)** with LDA score > 3 predicted using PICRUSt2. |LDA scores| > 3, *P* < 0.05. AH, adenoid hypertrophy; HCs, healthy controls; LEfSe, linear discriminant analysis effect size; LDA, linear discriminant analysis.

We then used linear discriminant analysis effect size (LEfSe) to identify the key distinctive features of the gut microbiota in AHs. Based on linear discriminant analysis (LDA), 23 genera were identified to be differentially abundant between these two groups at a relatively strict cutoff (LDA > 3, *p* < 0.05). Compared with HCs, nine genera, including *Bacteroides*, *Lachnospiraceae*, and *Ruminococcus gnavus*, were enriched in AH patients, whereas 14 genera, including *Akkermansia* and *Eubacterium_coprostanoligenes*, were significantly reduced (**Figure 3A**, **Supplementary Table 12**). In addition, a lower abundance of the *Proteobacteria* phylum, *Bacilli*, *Coriobacteriia*, *Verrucomicrobiae* classes, and *Christensenellales*, *Pseudomonadales* orders was found in AH children, while abundances of the *Negativicutes* class and *Bacteroidaceae* family were higher in AHs (**Figure 3B**). More information can be found in the supplementary data (**Supplementary Figure 6**, **Supplementary Table 12**). These results indicated that children with AH have significantly distinct microbial signatures.

### 3.5 Microbial functions changes related to AH

Phylogenetic Investigation of Communities by Reconstruction of Unobserved States 2 (PICRUSt2) and 16S rRNA gene sequences were used to predict the AH-related gut microbial functions. Using the Kyoto Encyclopedia of Genes and Genomes (KEGG) database, 32 modules showed significant differences between AH and HCs, of which 9 and 23 were enriched in AH patients and HCs, respectively (*p* < 0.05, LDA > 3; **Figure 3C**, **Supplementary Table 13**). Compared with HCs, metabolic systems including ribose transport system, phosphotransferase system (PTS) system, galactitol/glucitol sorbitol/mannitol specific II component, lipopolysaccharide (LPS), ATP binding cassette (ABC) transport system, and uronic acid were dramatically upregulated, while 5-phospho-D-ribosyl-α-1-diphosphate (PRPP) biosynthesis and nicotinamide adenine dinucleotide (NAD) biosynthesis-related modules were downregulated in AHs. The influenced pathways by the gut microbiota from AHs included the upregulation of porphyrin and chlorophyll metabolism and the downregulation of linoleic acid metabolism (*p* < 0.05, LDA > 3; **Figure 3D**, **Supplementary Table 13**).

### 3.6 Diagnostic model of AH based on the gut microbial markers

**Figure 4:**
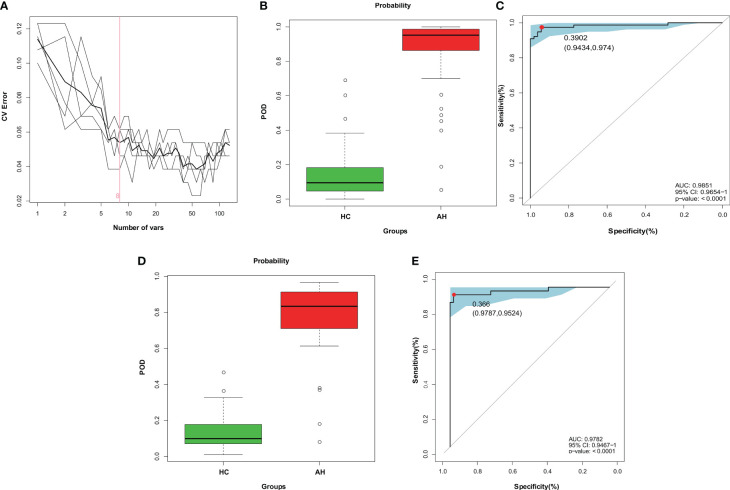
Diagnostic potential of gut microbial markers in AH patients based on random forest classifier model. **(A)** Identification of gut microbial markers by random forest classifier model. **(B)** Compared with HCs (n = 53), the POD value was higher in AH patients (n = 77) in the discovery cohort. **(C)** The POD index reached an AUC value of 0.9851 with 95% CI of 0.9654 to 1 between AHs (n = 77) versus HCs (n = 53) in the discovery cohort (*P* < 0.0001). **(D)** In the validation cohort, the POD value was higher in AH patients (n = 42) than in HCs (n = 47). **(E)** The POD index reached an AUC value of 0.9782 with 95% CI of 0.9476 to 1 between AHs (n =42) versus HCs (n = 47) in the validation cohort (*P* < 0.0001). AH, adenoid hypertrophy; HCs, healthy controls; POD, probability of disease; CI, confidence interval; AUC, area under the curve.

At present, the diagnosis of AH relies mainly on nasal endoscopy. The growing patient population demands the development of a more efficient and noninvasive technique. Hence, in this study, we constructed a diagnostic model for AH based on gut microbial markers. In the discovery cohort, 77 children with AH and 53 healthy controls living in a local region of East China (Zhejiang Province, Jiangsu Province, and Shanghai City) were used to construct a random forest classifier model. Among the 29 differentially abundant OTUs (**Supplementary Figure 7**), eight key OTUs were selected by fivefold cross-validation of the random forest classifier model to assess the diagnostic potential of gut microbial markers for AH using the area under the curve (AUC) (**Figure 4A**, **Supplementary Table 14**). The consistency of the results predicted by the decision trees based on these eight key OTUs and the actual disease condition was represented by the probability of disease (POD) index. The POD value of AH significantly increased compared to that of the HCs (*p* < 0.001, **Figure 4B**, **Supplementary Table 15**). The classification performance of the model was assessed with the area under the receiver operating characteristic (ROC) curve (AUC) of the POD index. In the discovery cohort, this microbial panel efficiently distinguished AH patients from healthy controls (AUC = 0.9851; *p* < 0.0001; **Figure 4C**), suggesting its potential for diagnosing AH.

To test the discriminative ability of these eight microbial markers, a validation cohort containing 42 AH patients and 47 healthy controls was recruited from other regions of China, including Gansu province in the Northwest, Tibet in the West and Guangdong province in the South of China (**Supplementary Table 16**). In the validation cohort, the POD value was observably higher in AHs than that in healthy control samples (*p* < 0.001; **Figure 4D**, **Supplementary Table 17**), and the AUC value was 0.9782, suggesting high accuracy in differentiating AH from HCs (*p* < 0.0001; **Figure 4E**). These data validated the remarkable potential of the classifier model based on gut microbial markers for the diagnosis of AH.

### 3.7 Associations of gut microbiota with AH clinical parameters

**Figure 5:**
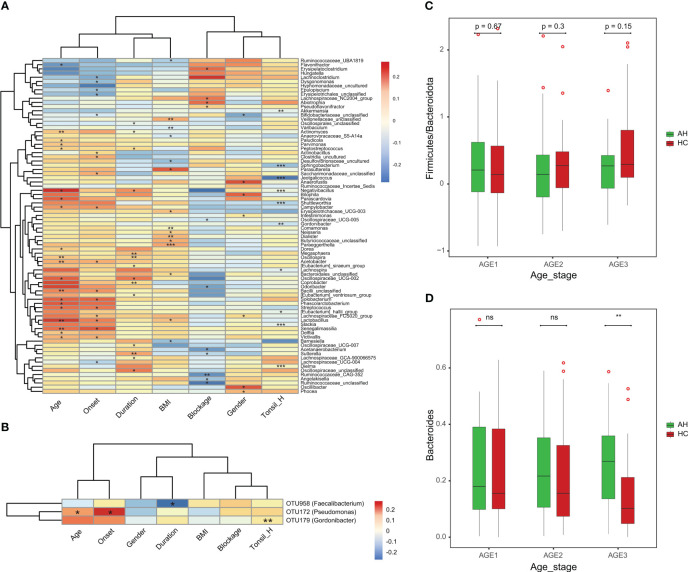
The associations of clinical parameters with the gut microbiota. **(A)** The heat map of Spearman’s rank correlation coefficients between the gut microbiota and clinical parameters (*P* < 0.05). *P < 0.05; **P < 0.01; ***P < 0.001. **(B)** The heat map of Spearman’s rank correlation coefficients between the eight OTU markers and clinical parameters (*P* < 0.05). *P < 0.05; **P < 0.01; ***P < 0.001. **(C)** The *Firmicutes*/*Bacteroidota* ratio changes with age in AH patients and HCs. **(D)** The abundance pattern of *Bacteroides* in different age stages in the AH patients and HCs. AH, adenoid hypertrophy; HCs, healthy controls.

Next, we assessed the associations between the clinical parameters and gut microbiota. At the genus level, BMI was negatively correlated with *Oscillospiraceae_UCG-007*. Male patients were positively correlated with a higher level of *Anaerofustis*. Moreover, the severity of the blockage was positively correlated with *Flavonifractor* but negatively correlated with *Bacilli_unclassified* (**Figure 5A**). Among the eight OTU markers selected by the classifier model, the onset of AH was positively correlated with OTU172 (*Pseudomonas*), whereas the duration of illness was negatively correlated with OTU958 (*Faecalibacterium*) (**Figure 5B**). We then divided the AH children and healthy controls into three groups according to age (Age1: 2–3 years old; Age2: 4–5 years old; Age3: 6–8 years old). The ratio of *Firmicutes* to *Bacteroidetes* in healthy children gradually increased with age. In contrast, this ratio remained constant in children with AH, although no significant differences were detected (**Figure 5C**). Interestingly, the abundance of *Bacteroides* changed with age in the AH and HC groups (**Figure 5D**). Significant differences could have been detected by the age stage of 6-8 years.

### 3.8 Gut microbiota from AH patients affects immune homeostasis in mice

**Figure 6:**
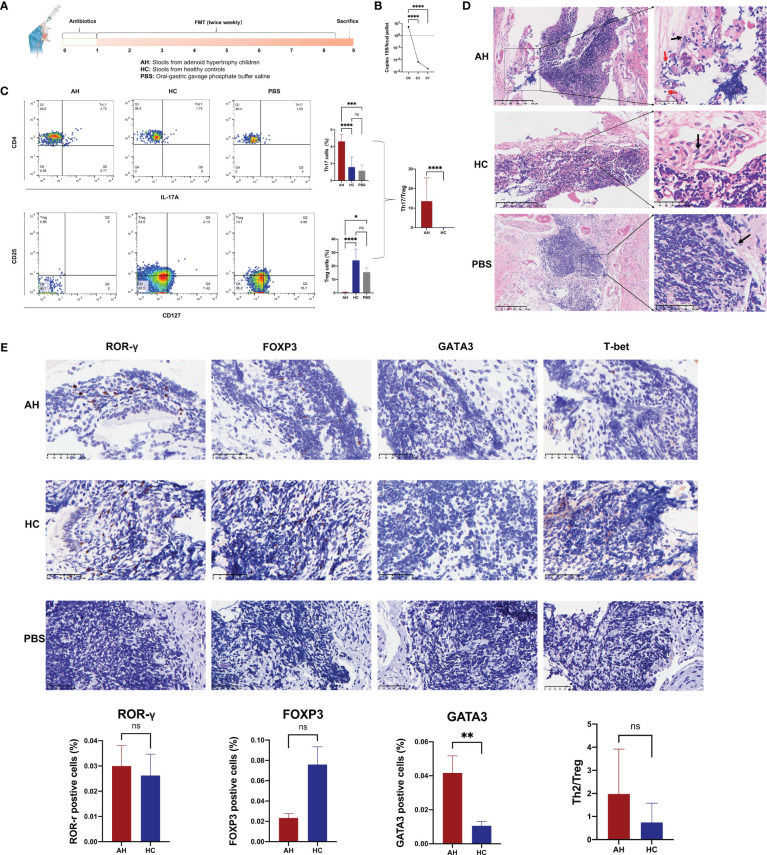
Gut microbiota in AH patients induce alternation of immune status in mouse NALT. **(A)** The scheme of experimental design for the fecal microbiota transplantation into mice. **(B)** The density of bacterial DNA copy number in mice feces during antibiotic treatment. ****, *P* < 0.0001. **(C)** The proportion of Th17 and Treg subsets were analyzed by flow cytometry and compared among AH, HC, and PBS groups. ****, *P* < 0.0001; ***, *P* < 0.001; *, *P* < 0.05. **(D)** H&E staining of NALT from mice subjected to FMT (red arrow: lymphocyte; black arrow: epithelial cell). **(E)** Immunohistochemical analysis of lymphocyte subtypes in NALT and the statistical comparison. AH, adenoid hypertrophy; HCs, healthy controls; NALT, nasal-associated lymphoid tissue; FMT, fecal microbiota transplantation; PBS, phosphate buffer saline. **, *P* < 0.01; ns, no significance.

To investigate the effect of gut microbiota on adenoid enlargement, 21 mice were orally administered bacterial suspension from the participant stool for eight weeks (**Figure 6A**). Before performing FMT, the mice were administered an antibiotic cocktail for seven days to reduce intestinal bacteria (**Figure 6B**). Similar with the results of participants, we found *Akkermansia* genera was significantly reduced in the mouse feces of AH group (**Supplementary Figure 8**). Flow cytometry analysis showed that, compared with other groups, the population of Th17 cells among the peripheral blood CD4^+^ T cells was significantly increased in the AH group [*p* < 0.0001 (HC), *p* < 0.001 (PBS); **Figure 6C**]. In contrast, the proportion of Treg cells in mice transplanted with microbial samples from AH individuals dramatically decreased [*p* < 0.0001 (HC), *p* < 0.05 (PBS); **Figure 6C**]. Compared with mice that received PBS only, the Th17 and Treg subsets in the HC group increased slightly without a significant difference. In addition, the Th17/Treg in AH group was significantly higher than HC group. No difference was found in the CD4/CD8 ratio among the three groups (**Supplementary Figure 9**). These results indicated that the proportion of T lymphocyte subsets in the peripheral blood of mice receiving FMT from pediatric patients with AH underwent pronounced changes, implying that the gut microbiota of AH patients may induce a systemic immune imbalance.

In the NALT of transplanted mice, we found that the gene expression level of TLR4 in AH group was significantly higher than that in HC group (**Supplementary Figure 10**). Histological examination showed that lymphocytes in the tissue of the AH group infiltrated the surrounding epithelial tissue (**Figure 6D**). We used ROR-γ to mark the Th17 subset in NALT. However, the number of ROR-γ+ Th17 cells increased only slightly. Nevertheless, the number of GATA3+ Th2 cells was significantly higher in the NALT of the AH group than that in the HC group (**Figure 6E**). In addition, FOXP3+ Treg cells increased more than three times in the NALT of the AH group compared to the HC group, although this difference was not statistically significant. Therefore, the ratio of Th2/Treg was ten-fold higher in the AH group than in the HC group. In addition, T-bet/Tbx21+ Th1 cells were not identified in the NALT of either group. These results suggested that dysregulation of the gut microbiota plays an important role in controlling allergic Th2 inflammation in the homologous organ of the adenoid.

## 4 Discussion

In recent years, it has been widely considered that disturbed normal development of the gut microbiome in children causes a series of chronic diseases, such as obesity (43), hypertension (44), and atherosclerotic disease (45). Adenoid hypertrophy is the most common cause of upper airway obstruction in children and adolescents. The local microecology of adenoid hypertrophy has been widely studied with sequencing-based methodology. For instance, *Bacteroides* and *Streptococcus* were found in the crypts of adenoid, *Fusobacteria*, *Pseudomonas* and *Burkholderia* were observed in adherent bacterial infiltrates and layers, and *Heamophilus influenzae* infiltrated in the epithelium of tissue (46). However, no consensus conclusion has been made so far, suggesting that other etiological mechanisms may also exist. Currently, an effective treatment approach for AH is adenoidectomy, indicated by overnight polysomnography and fiber nasopharyngoscopy (33). To date, gut microbiome has emerged as a new medical tool to prevent, diagnose, and treat various immune-related respiratory tract diseases, including AR and asthma. Compared with endoscopic diagnosis with low cooperation and surgical treatment with infection risk of antibiotic resistant bacteria, gut bacterial-markers may also be a novel method to diagnose and treat adenoid hypertrophy. However, few studies have examined the relationship between AH and the gut microbiome in children. In this study, we characterized the composition and function of the gut microbiome in children with AH compared to those in HCs. We also identified eight gut microbial markers that presented strong diagnostic potential to distinguish AHs from HCs. Although these results need to be validated further, they may also provide novel therapeutics for the clinical treatment of children with AH.

Our study comprehensively elucidated the effect of gut microbial profiling on AH in children using 16S rRNA sequencing. Herein, we found that only a slight decrease in the diversity could be detected by the Simpson index, which is revealed by the increase of the index value. Whereas no significant differences in α-diversity between AH and HCs were detected by the other three indices. Interestingly, it has been shown that children who developed asthma showed low diversity of gut microbiota (47), and the alteration of bacterial diversity in children with AR is still debated (22, 48). Thus, the unchanged α-diversity may be a specific feature for AH that is different from other allergic airway diseases.

As shown in the results, the gut microbial taxonomic composition and abundance of diverse bacteria in children with AH, were greatly different from HCs at phylum, class, order, family, and genus level. The abundance of two phyla, *Verrucomicrobiota* and *Patescibacteria*, significantly reduced in AH patients relative to HCs. *Verrucomicrobia* is a small phylum involved in nitrogen fixation. This phylum resides in the mucous lining of the intestinal tract with high abundance in healthy individuals, suggesting their roles in glucose homeostasis of the human gut (49). *Verrucomicrobia* phylum are also positive correlated with the expression of FOXP3 and can be essential to a healthy gut due to its anti-inflammatory properties (50). At the genus level, 28 genera bore remarkable advantages in AHs while 76 genera were significantly dominant in HCs. It has been reported that children with a relatively high abundance of *Bacteroides* genus and low abundance of *Bifidobacteria*, *Akkermansia* and *Ruminococcus gnavus* had a greater risk of developing asthma and allergic rhinitis (51). In consistent with our data showing that *Bacteroides* had a marked increase and *Akkermansia* decreased in the gut of AH patients. Moreover, as the most predominant genus, the abundance of *Bacteroides* in AHs appears constantly elevated with age, in an opposite trend to that in HCs. However, *Ruminococcus gnavus* was significantly higher in the AHs. It was evident that the cell wall of *Ruminococcus gnavus* contained glucorhamnan. When the abundance of *Ruminococcus gnavus* increased, the polysaccharide stimulated the secretion of proinflammatory cytokines in a TLR4-dependent manner in the intestinal epithelial cells (52, 53), thus, it may play an important role in the immune response. Among the reduced bacterial genera, *Streptococcus* is commensal in the human gut, which can attenuate the mucosal proinflammatory state induced by LPS (54). *Eubacterium hallii* is an important butyrate producer. This lipid has been shown to induce the expression of FOXP3 by inhibiting histone deacetylation, resulting in systemic anti-inflammatory properties via the expansion of Tregs (21), which was reported to be significantly lower in patients with allergic rhinitis (48). Similar reduction pattern between AH and allergic rhinitis was also observed for *Romboutsia*, *Collinsella* and *Dorea*. *Oscillospira* is an understudied genus of anaerobic bacteria. Recent evidence has shown that *Oscillospira* was enriched in lean subjects for degrading fibers and decreases in abundance in inflammatory diseases, probably involved in SCFAs production (55). *Akkermansia* belongs to *Verrucomicrobia* phylum, which inhabits the human gut intestinal tract for mucin degradation. Numerous studies have indicated that *Akkermansia* played an important anti-inflammatory role in the progression of diseases such as obesity (20, 56). As in the case of AH, reduced *Akkermansia* may lose its ability to protect the intestinal barrier, leading to systemic inflammation.

The effects of the gut microbiota on pathology of the other organs are mainly mediated by bacterial metabolites, which may influence immune responses in distal parts of the body (57). Compared to HCs, the linoleic acid metabolism was disrupted in children with AH. Linolenic acid can improve the bacterial community, intestinal wall barrier, and inflammatory environment, and reduce the level of LPS in mice (58). In contrast, porphyrin and LPS metabolism were enhanced in AHs. Porphyrins are intermediate metabolites involved in the biosynthesis of vital molecules, including cobalamin. Bacterial porphyrins are known to be proinflammatory (59). LPS can act locally and systemically after crossing the gut barrier and entering circulation. Elevated concentrations of LPS in peripheral blood were associated with chronic immunological diseases (60). In our study, the biological metabolic function of ribose and the carbohydrate transport system, such as the PTS system and ABC transport system, were greatly active in AH, while the metabolism of PRPP was significantly decreased. A previous study demonstrated that the polysaccharide-utilization loci (PUL) of *Bacteroides* was a symbol of a ribose-utilization system (61). *Bacteroides* spp. could then induce the secretion of IL-6, which is a pivotal issue in NF-κB-induced inflammatory and induction of STAT3 signaling to promote Th17 responses (62–66). The activation of the PTS system, extensively distributed in *Eubacteria*, inhibited aromatic amino acid biosynthesis, similarly, the reduction of PRPP also suppressed the biosynthesis of tryptophan (67–70). The reduction of kynurenine and indole, endogenous and bacterial metabolites of tryptophan, respectively, activated the production of proinflammatory chemokines and NF-κB (71–73). These results suggested that the alterations in gut microbiome function in AH may be associated with the production of metabolites.

Notably, our study is the first to show that AH patients can be distinguished from HCs by their gut microbiota. We selected eight optimal microbial markers to distinguish between patients with AH based on the random forest model. The model not only achieved satisfactory classification efficacy but also had strong diagnostic potential. More importantly, we also conducted geographical validation of gut microbial marker-based AH classifiers. Previous studies have shown that geographical distance and diet were the predominant influences on gut microbiome variation (74, 75). We used samples from AH children who lived in East China, including Zhejiang Province, Jiangsu Province, and Shanghai City, as the discovery set and achieved a high accuracy (AUC = 0.9851). Populations living in these areas appear to be a common culture in the South Yangzi River and have very similar dietary habits. A validation set comprising children with AH from other regions achieved an AUC of 0.9782. These subjects came from areas such as Sichuan province where chili peppers are preferred food, Guangdong province where seafood is popular, and Yunnan and Tibet, where ethnic minorities live. The performance of the AH classifier model revealed that despite the influence of diet and genetic background on gut microbial variation, gut microbiome-targeted biomarkers have the potential to be used as noninvasive tools for AH in the future. *Firmicutes* and *Bacteroidetes* are the two major bacterial phyla constituting 90% of the total microbial community. *Firmicutes* to *Bacteroidota* (F/B) ratio was shown to significantly increase throughout the life course (76). Imbalance in F/B ratio was frequently reported in various chronic diseases (77). Indeed, this ratio did not increase with age as in healthy children, but remained at a stable level in AH patients, which may suggest that the intestinal microbiota of AH children is in a relatively fixed state without changing over time. Therefore, gut microbiota-targeted biomarkers can potentially be used to predict the occurrence of AH. Nevertheless, this model needs to be validated in a prospective study with a larger cohort.

The mechanisms mediating communication between the gut and distal organs are still under investigation, but it has been suggested that the immune system is one of the most critical ways to stimulate responses at distal sites (78, 79). We found that consistent with previous reports in children with AH (27, 80), Th17 cells significantly increased while Treg cells dramatically decreased in the peripheral blood of mice subjected to FMT from AH patients. Thus, the ratio of Th17/Treg subsets significantly elevated compared with that in mice transplanted with bacteria from HCs, suggesting that gut microbial dysbiosis in AH patients may lead to the dysregulation of systemic immunological processes. However, the number of tissue-resident Th17 cells in the AH group was slightly higher than that in the HC group. Instead, tissue-resident Th2 subsets drastically increased in the NALT of the AH group, while Treg cells reduced, leading to a disrupted Th2-Treg balance. Interestingly, in children with adenotonsillar regrowth, more GATA3+ Th2 cells and fewer FoxP3+ Treg cells were also observed at all ages (28). Although it is generally assumed that AH is associated with inflammation caused by recurrent respiratory infections and local pathogen stimulation, whether tissue can retract after recovery from acute infection varies between individuals. The recurrence and persistence of AH imply that Th2-represented allergic reactions may play a greater role in the development of the disease than the Th17 inflammatory response that responds to pathogen infection. Our results indicated that dysbiosis of the intestinal microbiota can cause changes in the allergic status of adenoids, possibly driving AH. This may also be closely related to the tendency of AH patients to accompany allergic rhinitis and develop asthma (81), in which Th2 cells hold a central position in the pathogenesis (82). The expansion of the Th2 subset may be due to the drastic reduction of Treg cells systemically and locally. Previous studies found that SCFAs produced by gut microbiota are important immunomodulators that increase the expression of the transcription factor FOXP3 via inhibition of histone deacetylation to induce the differentiation of Treg cells (83–85). *Eubacterium hallii*, *Oscillospira* and *Akkermansia* in the gut are involved in the production of SCFAs, primarily acetate, propionate, and butyrate. Therefore, the decreased abundance of these bacteria in the gut microbiota of children with AH may enhance susceptibility to inflammation by suppressing Treg cells, leading to long-lasting pathological symptoms. After eight weeks of FMT, we found that the colonization of *Akkermansia* genera dramatically decreased in mice transferred with microbiota suspension from patients with AH, which is accompanied by the increased gene expression of TLR4 in the NALT tissue of mice. Numerous studies have indicated that *Akkermansia* is involved in the regulation of various chronic diseases (86–88). *Akkermansia* protects the intestinal mucus layer and maintains intestinal homeostasis, reducing the entry of LPS into the bloodstream (89). Vesicles secreted by *Akkermansia* are also able to reduce the expression of TLR4, thereby regulating the NF-κB pathway (90). Therefore, insufficient *Akkermansia* genera might disrupt the balance of Th17/Treg through activating TLR4 signaling pathway. Details of the *Akkermansia*-specific regulatory mechanisms for AH will be intensively investigated in our future studies.

There were still several limitations worth to be discussed. First, we examined the changes of NALT of mice after eight weeks of fecal microbiota transplantation, when the mice were 12-week-old. According to Dutta et al’s estimation (91) and the guideline of the Jackson lab (https://www.jax.org/research-and-faculty/research-labs/the-harrison-lab/gerontology/life-span-as-a-biomarker), the transplanted mice at the experimental endpoint are approximately equivalent to the 20-year-old adolescents. Since AH in human usually occurs in children under six years old, we did not prolong our observation to the adult in mice, although longer transplantation may induce more drastic response. Whether this time point is suitable should be proved by time series experiments. Second, due to the limitation of the dietary information of children in this study, we cannot exclude the influence of diet habits. That means the uniqueness of patients’ interests on diet might play the driving role for AH. The microbiome differences in AH may only reflect their dietary preferences as argued for the potentials of the gut microbiome to autism spectrum disorder (92). Nevertheless, the distinct immunological changes that FMT mice displayed under the same growing conditions still suggest the contributions derived from the gut microbiome to the regulation of AH.

In this study, we characterized the different gut microbial compositions and functions of AH compared to those of HCs. In addition, we provided a gut microbial marker-based noninvasive diagnostic tool for distinguishing between AH and HC subjects and validated its efficiency cross-regionally. The predictive potential of this tool for AH occurrence and postoperative recurrence is implied in our data and requires further follow-up. Furthermore, the causal effect of intestinal dysbiosis on the balance of adenoid immunity was preliminarily demonstrated. Our study provides new insights into AH pathogenesis and provides novel directions for the treatment of adenoid hypertrophy in children.

## Data availability statement

All 16S rRNA gene sequencing data were deposited in the National Center for Biotechnology Information sequence read archive (SRA) repository, accession number:PRJNA893900.

## Ethics statement

The studies involving humans were approved by Shanghai Children’s Hospital Ethics Review Committee. The studies were conducted in accordance with the local legislation and institutional requirements. Written informed consent for participation in this study was provided by the participants’ legal guardians/next of kin. The animal study was approved by Shanghai Children’s Hospital Ethics Review Committee. The study was conducted in accordance with the local legislation and institutional requirements.

## Author contributions

WL: Writing – original draft, Writing – review & editing, Data curation, Methodology, Formal analysis. HJ: Resources. XLL: Software, Methodology. YZ: Methodology, Software. YL: Software, Methodology. FP: Investigation. FY: Investigation. ZL: Methodology. MG: Resources. QD: Conceptualization. XYL: Visualization. HZ: Funding acquisition, Project administration, Supervision. DH: Project administration, Supervision, Validation, Writing – review & editing.
