# Supplementary material for: Altered intestinal microbiota enhances adenoid hypertrophy by disrupting the immune balance
Source: Front Immunol. 2023 Nov 28;14:1277351. doi: 10.3389/fimmu.2023.1277351 (PMC10715246; doi:10.3389/fimmu.2023.1277351)
Supplement: Supplementary file 1 [file DataSheet_1.zip › Gut Microbiota Signature in Children with Adenoid Hypertrophy3-hdd-Supplementary Information/Supplementary Information.docx]

# Supplementary Materials

Supplementary Figures

**Supplementary Figure 1**. The serum level of twelve inflammatory in AH (n=30) and HC (n=30). AH, adenoid hypertrophy; HCs, healthy controls.

**Supplementary Figure 2**. The rarefaction curve indicated that the number of OTUs approaching saturation in AHs (n=119) and HCs (n=100) along with the increase of samples. AH, adenoid hypertrophy; HCs, healthy controls; OTUs, operational taxonomic units.

**Supplementary Figure 3**. Species accumulation curve indicating the saturation of sequencing reads in AHs (n=119) and HCs (n=100). AH, adenoid hypertrophy; HCs, healthy controls.

**Supplementary Figure 4**. The β-diversity composition of gut microbiome between AH (n=119) and HC (n=100). (**A**) The NMDS analysis indicating the difference of β-diversity between AH (n=119) and HC (n=100). (**B**) The CAP analysis indicating the consistency between of β-diversity and group classification based on diseases. AH, adenoid hypertrophy; HCs, healthy controls; NMDS, non-metric multidimensional scaling analysis; CAP, canonical analysis of principal coordinates.

**Supplementary Figure 5**. The differences in gut microbial composition between AH (n=119) and HC (n=100) at class level (**A**), order level (**B**), and family level (**C**). AH, adenoid hypertrophy; HCs, healthy controls.

**Supplementary Figure 6**. LEfSe analysis based on LDA effect size demonstrated differences in taxonomic composition of AH patients (n = 119) compared to HCs (n = 100). |LDA scores| > 2, *P* < 0.05. AH, adenoid hypertrophy; HCs, healthy controls; LEfSe, linear discriminant analysis effect size; LDA, linear discriminant analysis.

**Supplementary Figure 7**. Heatmap of the 29 different OTUs constructing a random forest classifier model.

**Supplementary Figure 8**. The relative abundance of *Akkermansia* genera in mouse feces between AH and HC group. AH, adenoid hypertrophy; HC, healthy control.

**Supplementary Figure 9**. The ratio of CD4/CD8 in the plasma in mice transplanted with AH, HC and PBS. AH, adenoid hypertrophy; HC, healthy control.

**Supplementary Figure 10**. The gene expression of TLR4 in the NALT of mouse between AH and HC group. AH, adenoid hypertrophy; HC, healthy control.

**Supplementary figure 1.** Twelve inflammatory cytokines in AH (n=30) and HC (n=30). AH, adenoid hypertrophy; HCs, healthy controls.

**Supplementary Figure 2.** A rarefaction curve between the number of OTUs and the number of samples in AH (n=119) and HC (n=100).

**
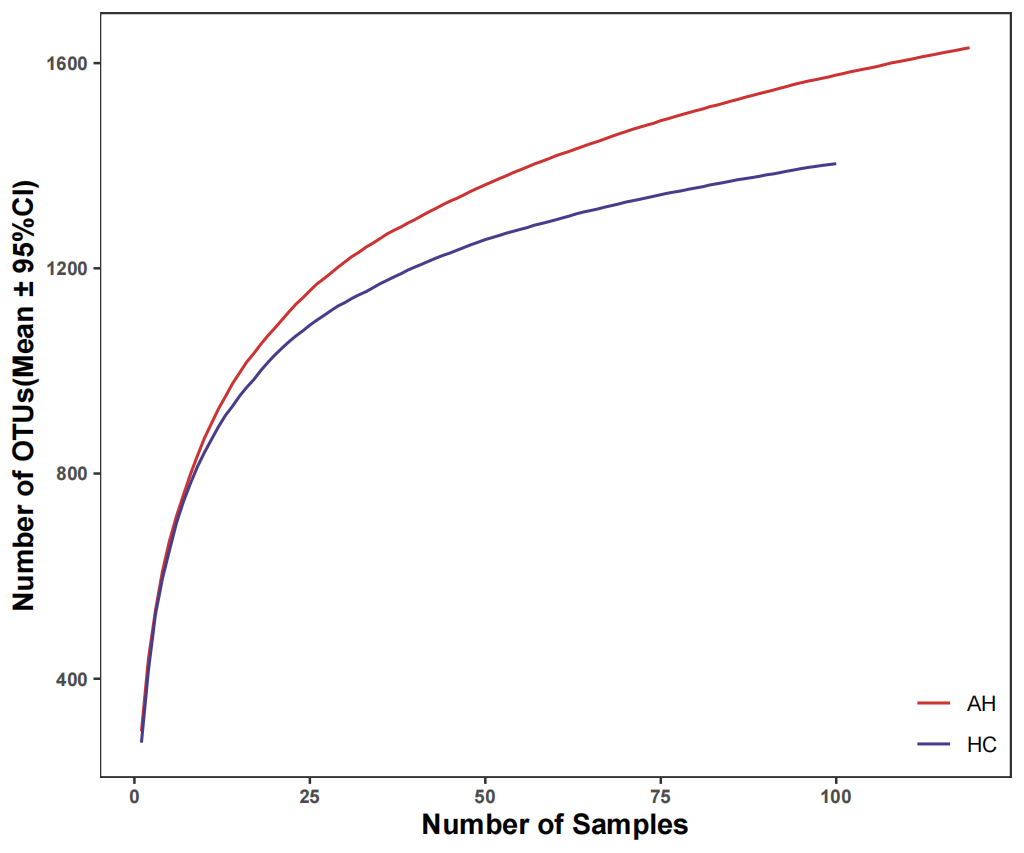
**

**Supplementary Figure 3.** Species accumulation curve indicating the number of samples in AH (n=119) and HC (n=100).

**
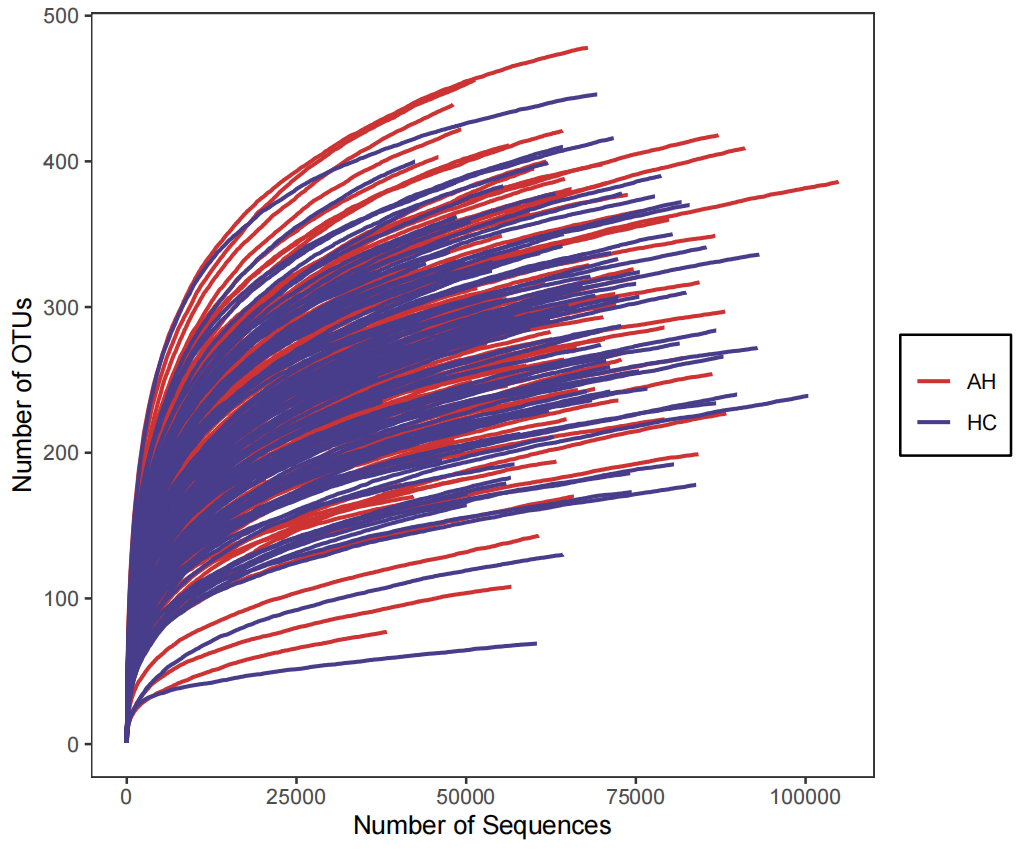
**

**Supplementary Figure 4.** The β-diversity composition of gut microbiome between AH (n=119) and HC (n=100).

**
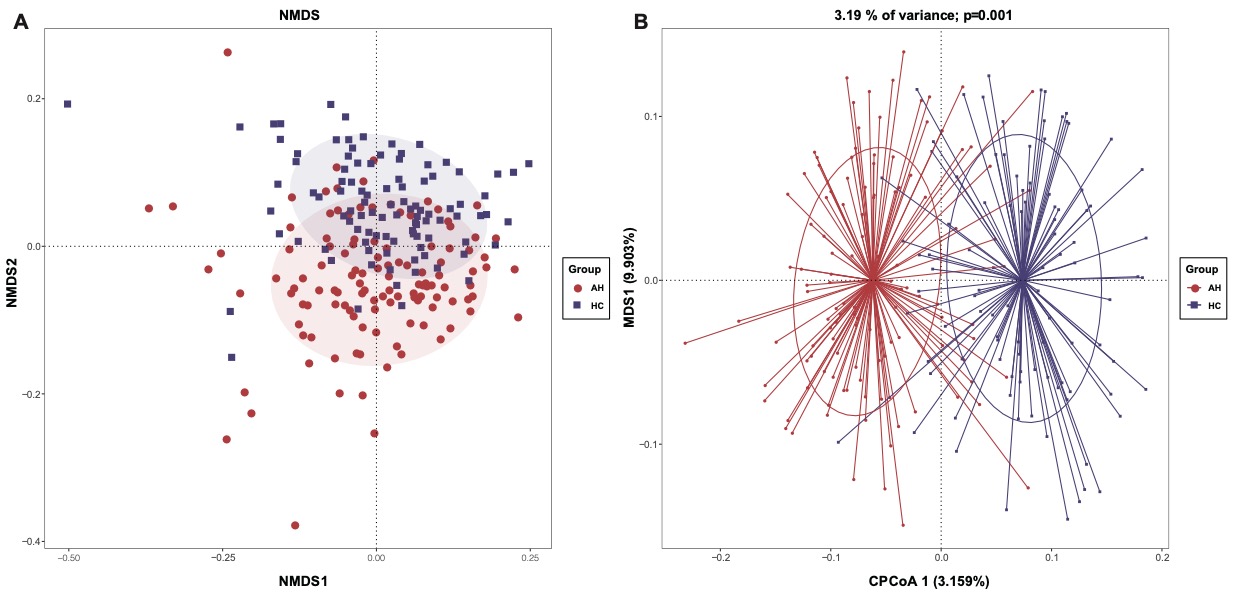
**

**Supplementary Figure 5.** The gut microbial composition between AH (n=119) and HC (n=100) at class, order and family levels.

**Supplementary Figure 6.** LEfSe analysis based on LDA effect size demonstrated differences in taxonomic composition of AH patients (n = 119) compared to HCs (n = 100).

**
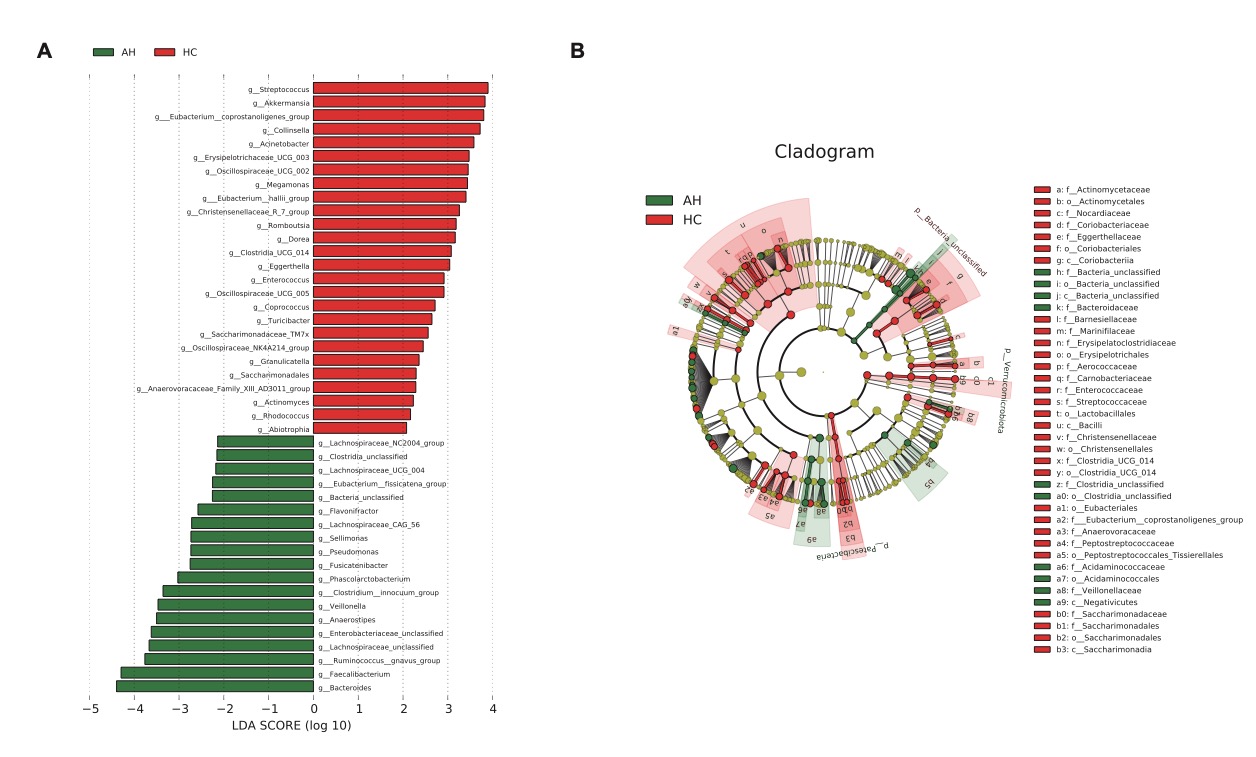
**

**Supplementary Figure 7.** Heatmap of the twenty-nine different OTUs constructing a random forest classifier model.

**
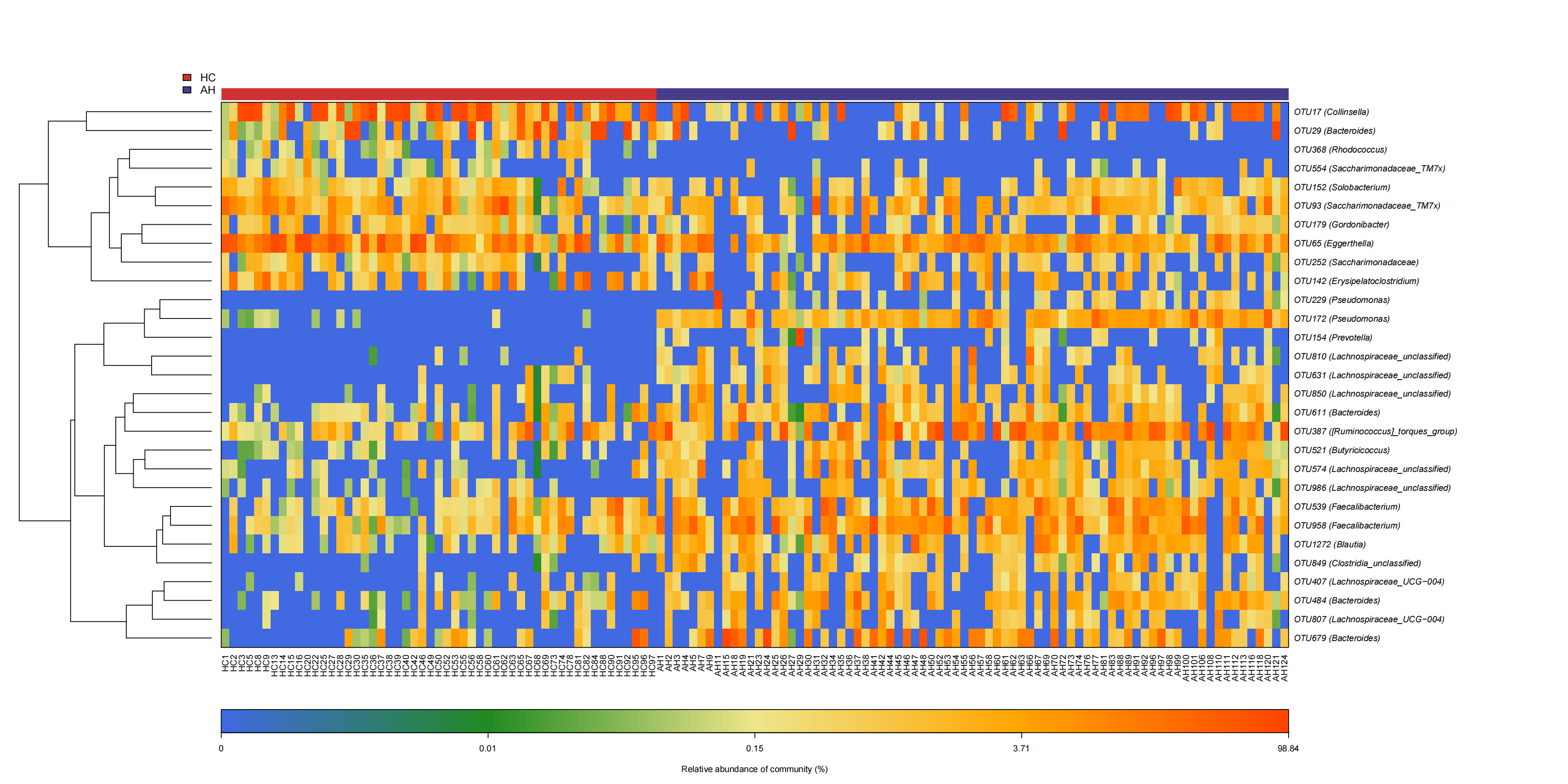
**

**Supplementary Figure 8.** The relative abundance of specific bacteria in mouse feces between AH and HC group.

**Supplementary Figure 9.** The plasma ratio of CD4/CD8 among the three groups.

**Supplementary Figure 10.** The gene expression of TLR4 in the NALT of mouse between the AH and HC group.

Supplementary Tables

**Supplementary Table 1**. Clinical characteristics of AH (n=119) and HC (n=100) individuals.

**Supplementary Table 2**. The serum level of inflammatory cytokines in participants from AH group (n=30) and HC group (n=30).

**Supplementary Table 3**. Statistics of valid sequences in fecal samples.

**Supplementary Table 4**. The α-diversity indices of gut microbiota and observed OTUs in AH (n=119) and HC (n=100) individuals.

**Supplementary Table 5**. The gut microbial composition of AHs (n=119) and HC (n=100) individuals at the phylum level.

**Supplementary Table 6**. The gut microbial composition of AH (n=119) and HC (n=100) individuals at the genus level.

**Supplementary Table 7**. The differential gut microbial composition between AH (n=119) and HC (n=100) individual at the phylum level.

**Supplementary Table 8**. The differential gut microbial composition between AH (n=119) and HC (n=100) individuals at the genus level.

**Supplementary Table 9**. The differential gut microbial composition between AH (n=119) and HC (n=100) individuals at the class level.

**Supplementary Table 10**. The differential gut microbial composition between AH (n=119) and HC (n=100) individuals at the order level.

**Supplementary Table 11**. The differential gut microbial composition between AH (n=119) and HC (n=100) individuals at the family level.

**Supplementary Table 12**. The LDA value (>2) and *p* value of the gene enrichment for AH (n=119) patients and HC (n=100).

**Supplementary Table 13**. The LDA value (>2) and *p* value of gene function enrichment for AH (n=119) and HC (n=100) gut microbial community.

**Supplementary Table 14**. The relative abundance of eight optimal microbial markers selected through random forest classifier model in the discovery cohort (77 AH patients and 53 healthy controls).

**Supplementary Table 15**. The POD value for each sample in the discovery cohort (77 AH patients and 53 healthy controls).

**Supplementary Table 16**. The relative abundance of eight optimal microbial markers selected through random forest classifier model in the validation cohort (42 AH patients and 47 healthy controls).

**Supplementary Table 17**. The POD value for each sample in the validation cohort (42 AH patients and 47 healthy controls).
