# Supplementary figures and images for: Altered intestinal microbiota enhances adenoid hypertrophy by disrupting the immune balance
Source: Front Immunol. 2023 Nov 28;14:1277351. doi: 10.3389/fimmu.2023.1277351 (PMC10715246; doi:10.3389/fimmu.2023.1277351)

# Akkermansia

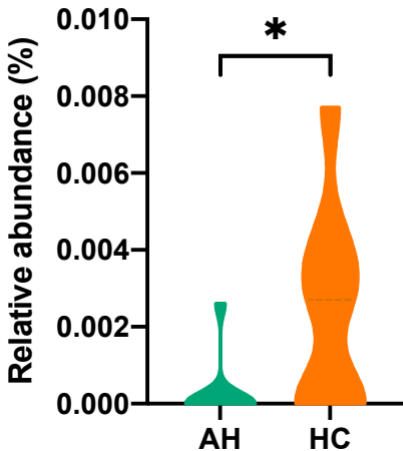

Supplement: Supplementary file 1 [file DataSheet_1.zip › Gut Microbiota Signature in Children with Adenoid Hypertrophy3-hdd-Supplementary Information/Supplementary Figure 8.pdf]

**CD8**

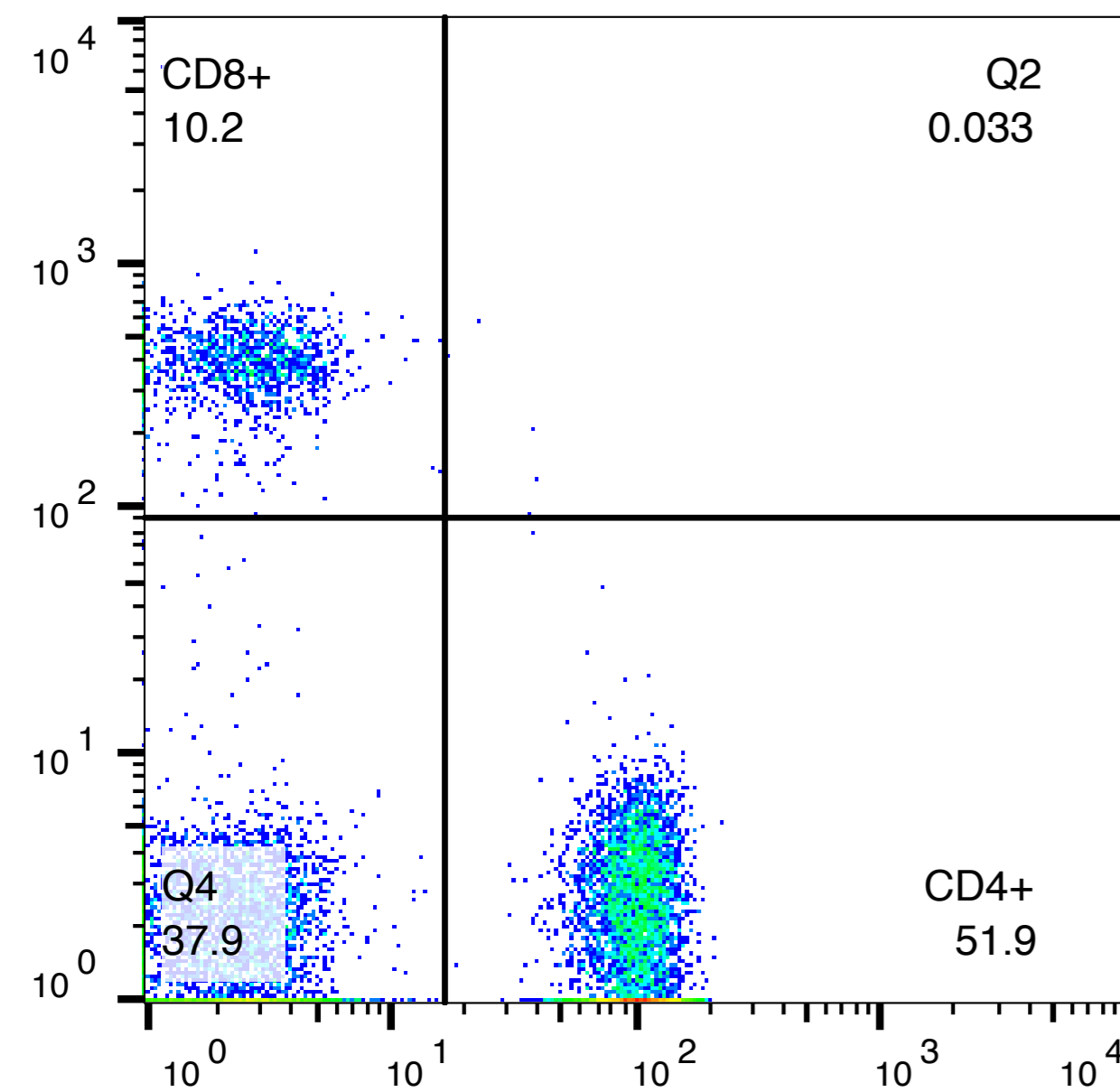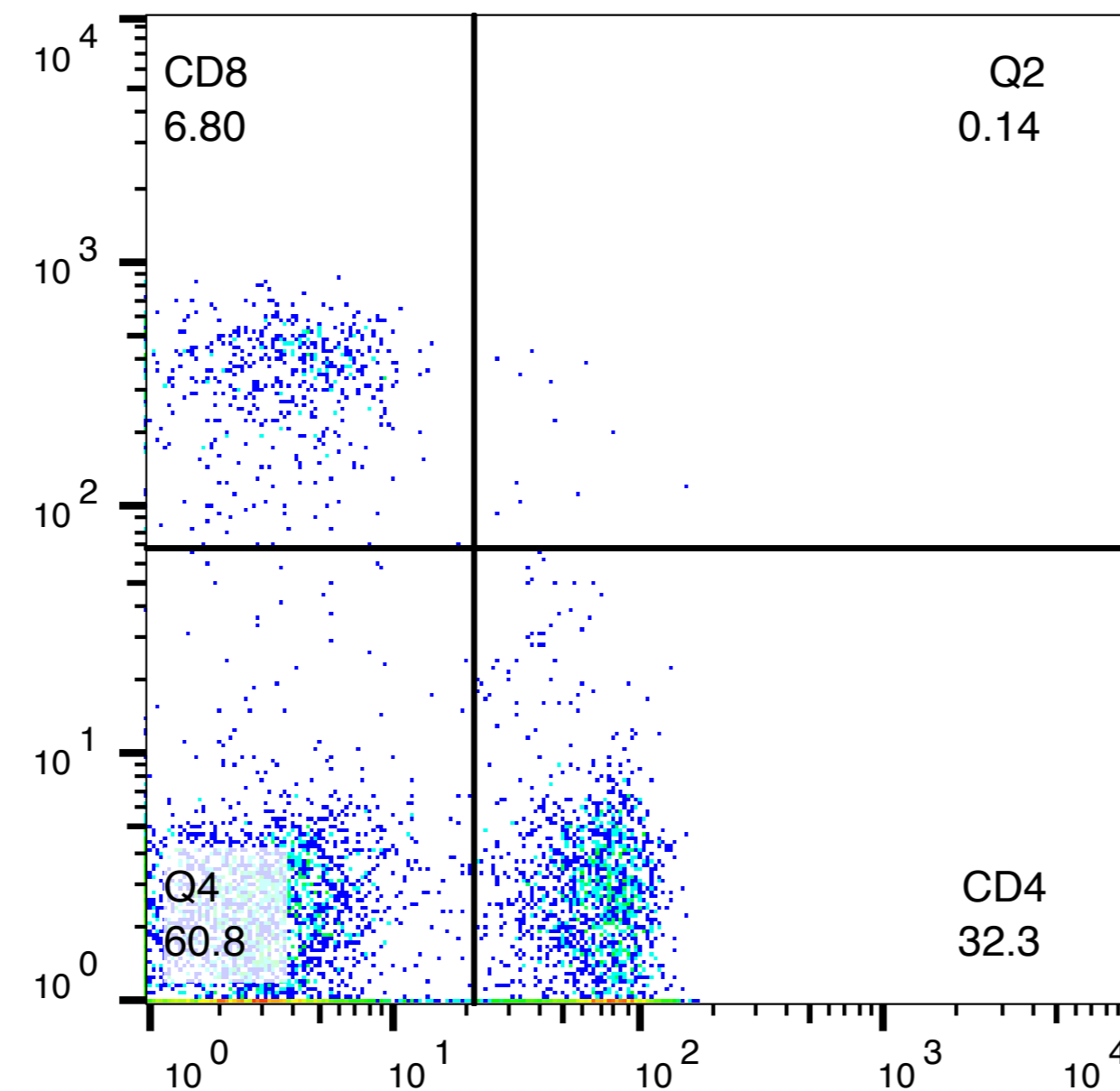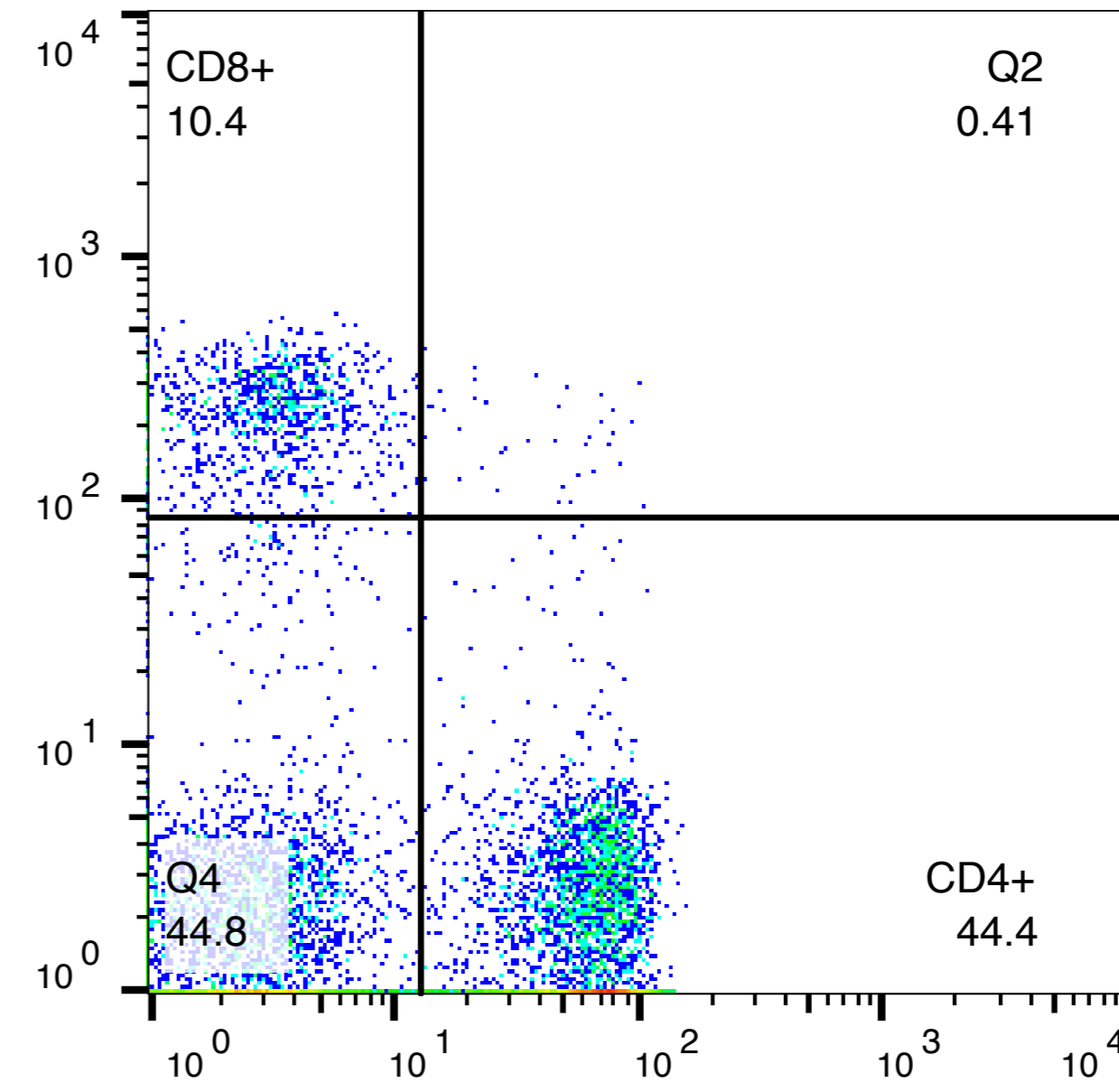

**CD4+/CD8+ cells**

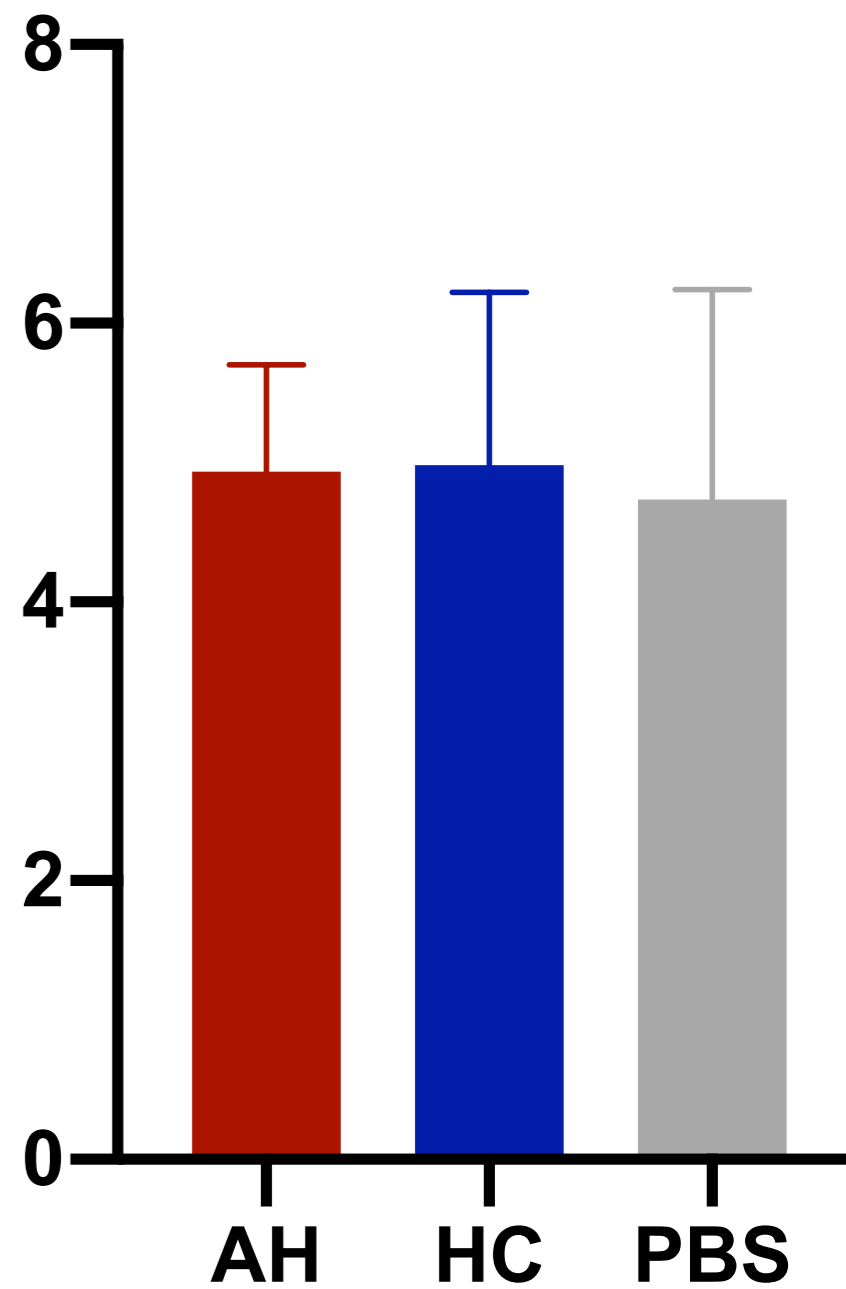

**CD4**

Supplement: Supplementary file 1 [file DataSheet_1.zip › Gut Microbiota Signature in Children with Adenoid Hypertrophy3-hdd-Supplementary Information/Supplementary Figure 9.pdf]

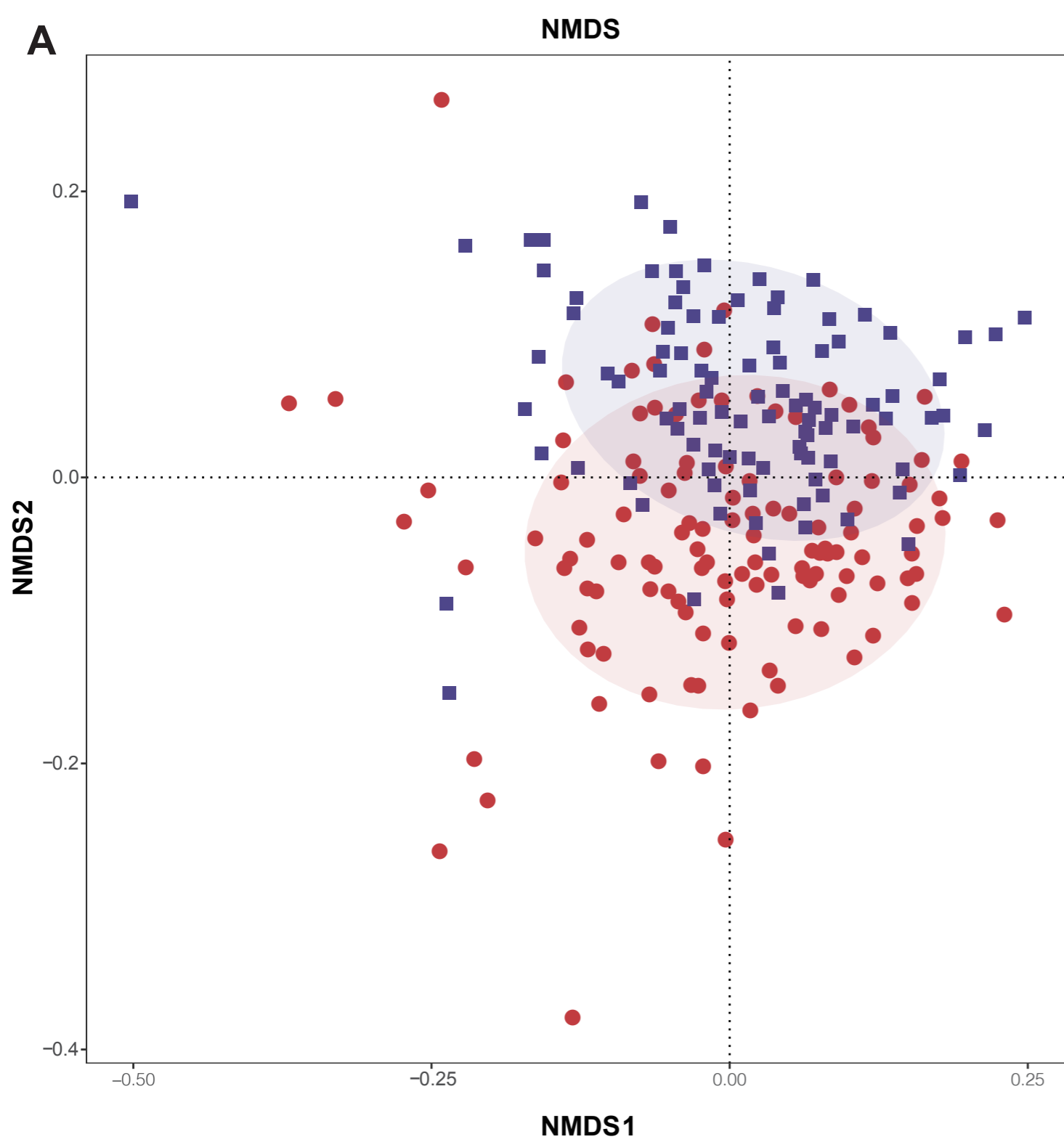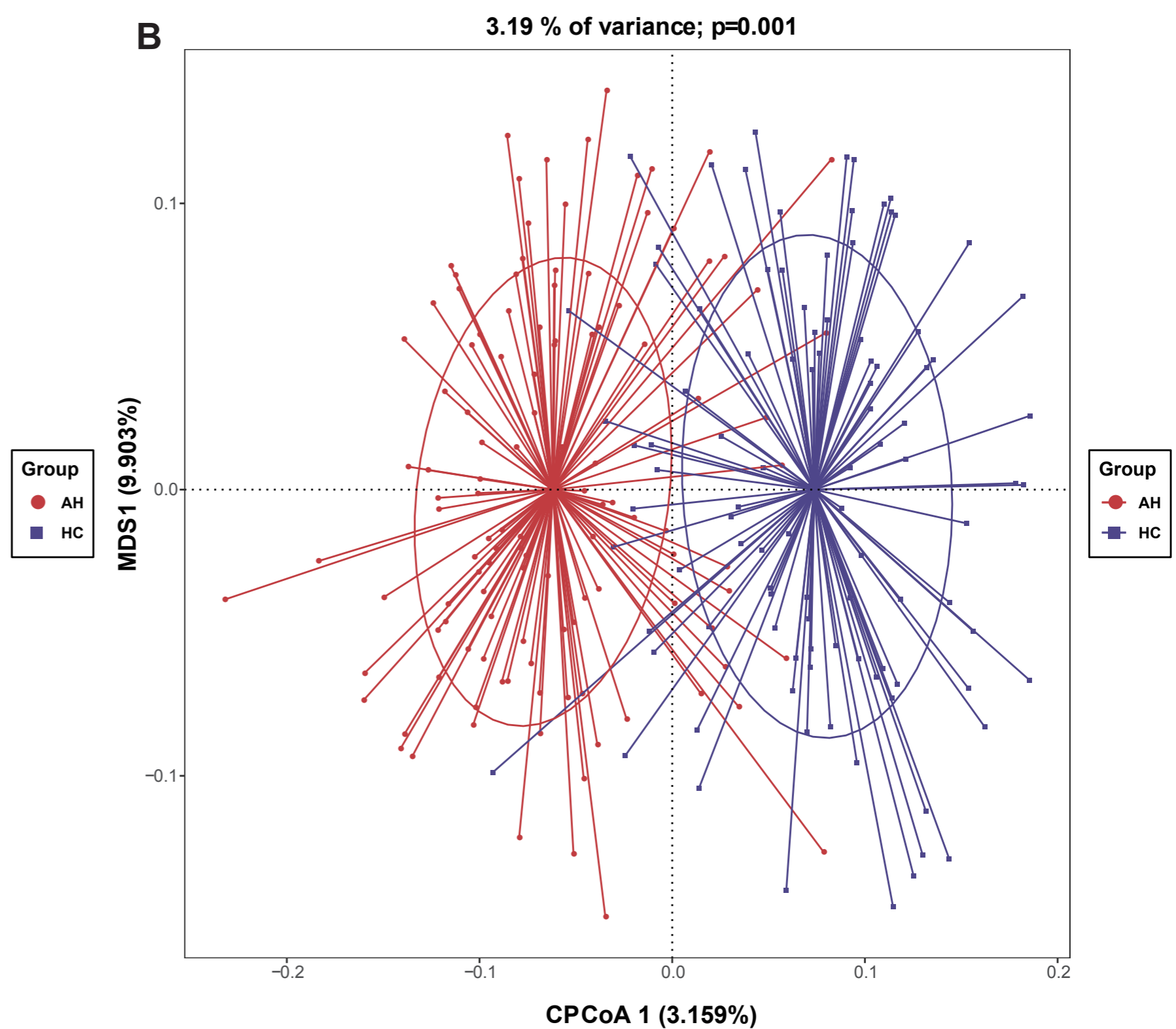

Supplement: Supplementary file 1 [file DataSheet_1.zip › Gut Microbiota Signature in Children with Adenoid Hypertrophy3-hdd-Supplementary Information/Supplementary Figure 4.pdf]

A

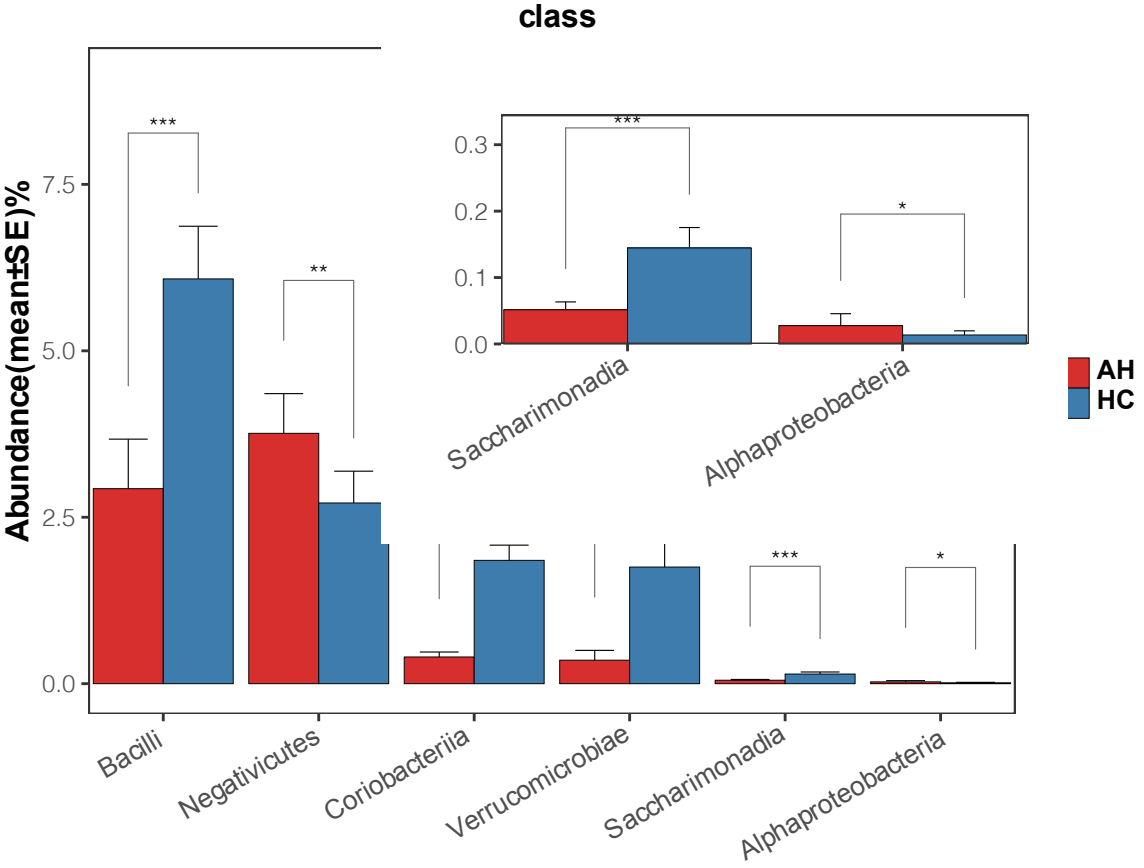

B

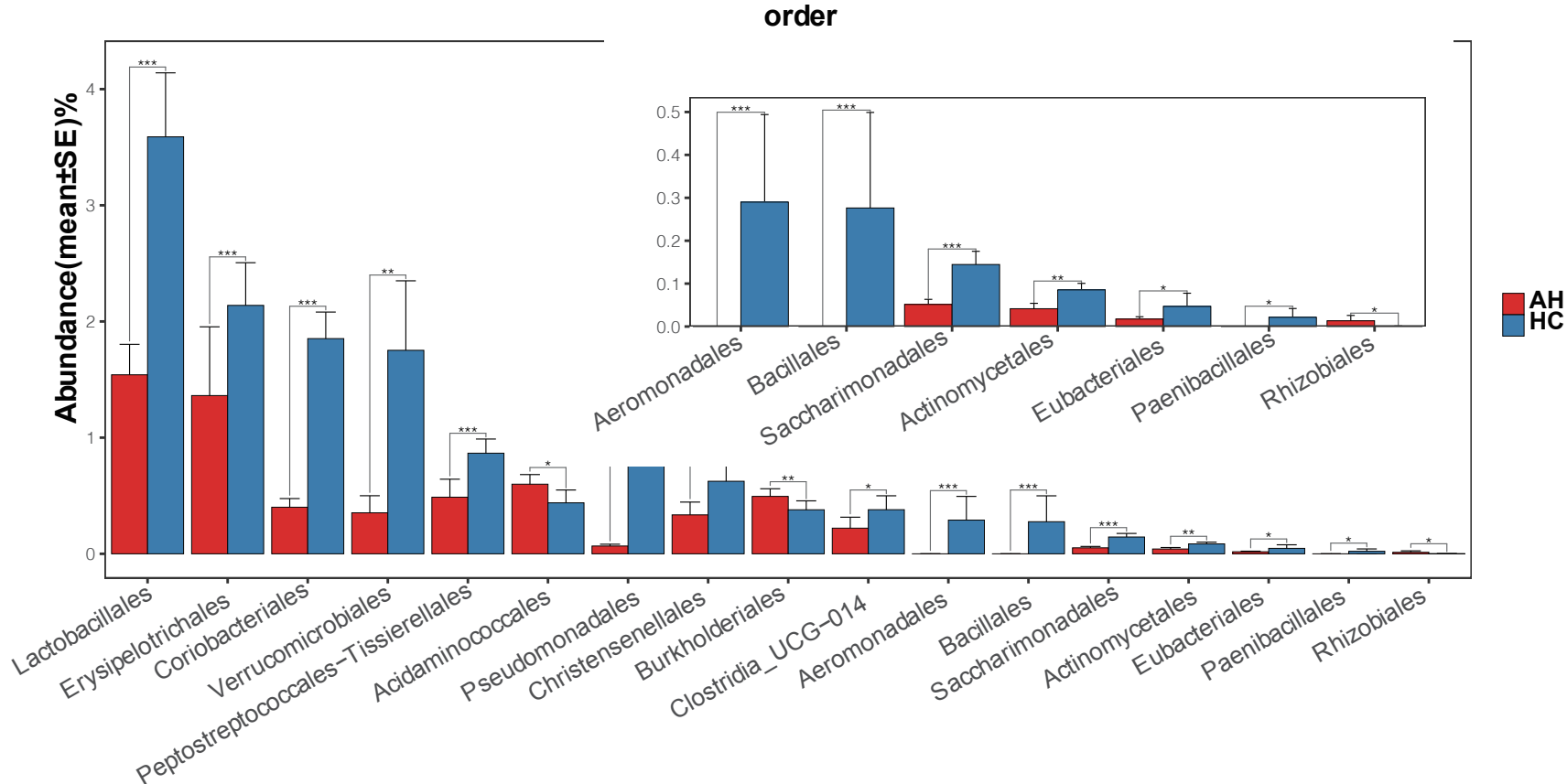

C

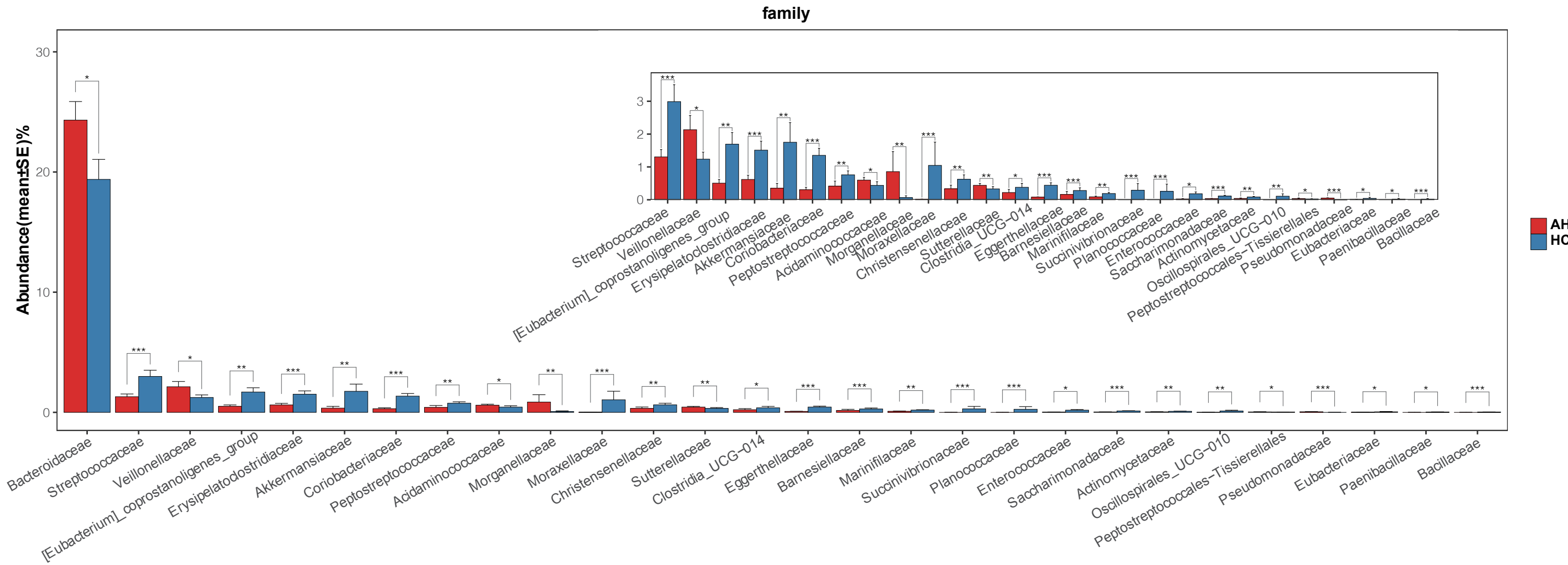

Supplement: Supplementary file 1 [file DataSheet_1.zip › Gut Microbiota Signature in Children with Adenoid Hypertrophy3-hdd-Supplementary Information/Supplementary Figure 5.pdf]

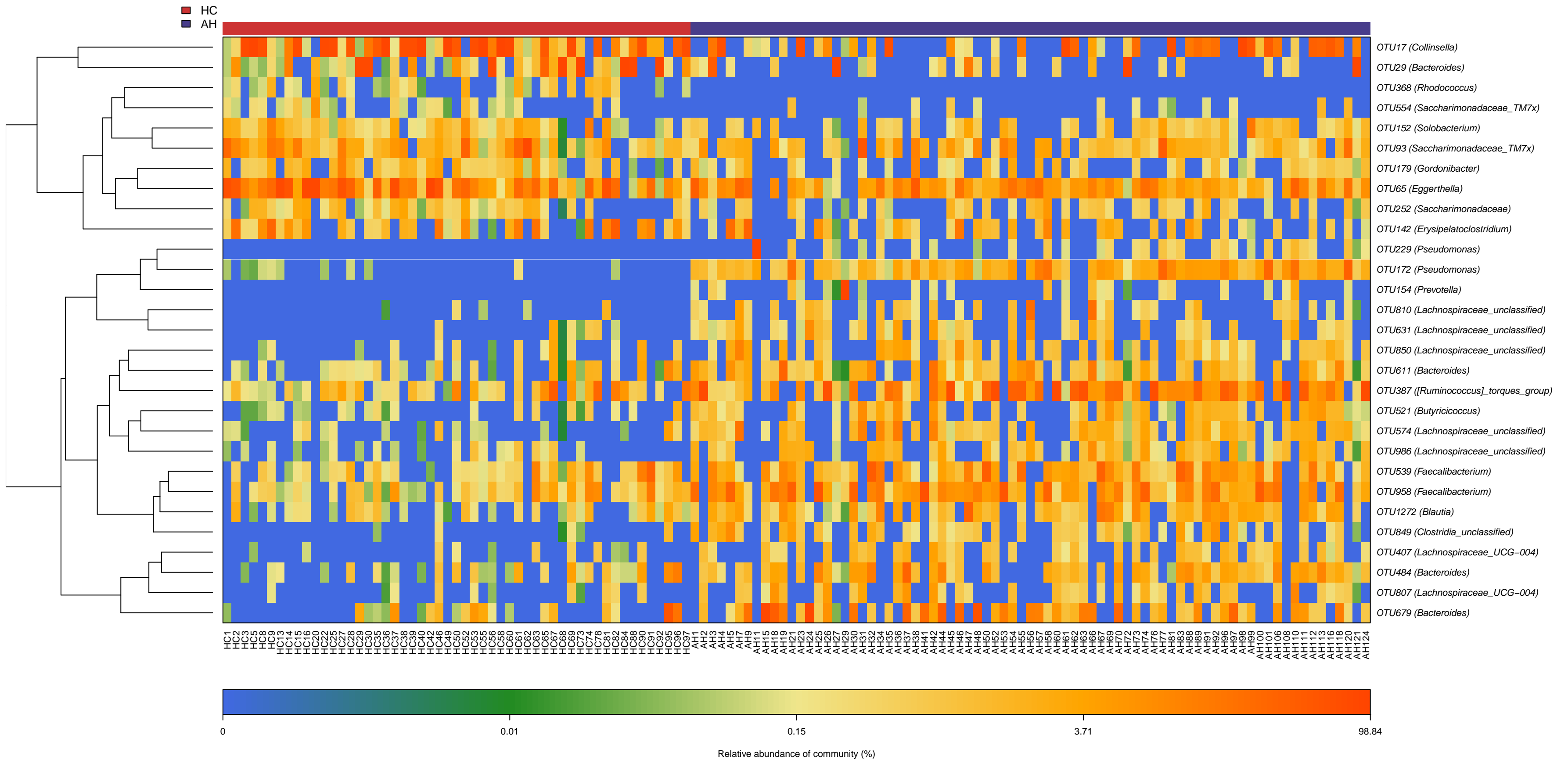

Supplement: Supplementary file 1 [file DataSheet_1.zip › Gut Microbiota Signature in Children with Adenoid Hypertrophy3-hdd-Supplementary Information/Supplementary Figure 7.pdf]

A

AH HC

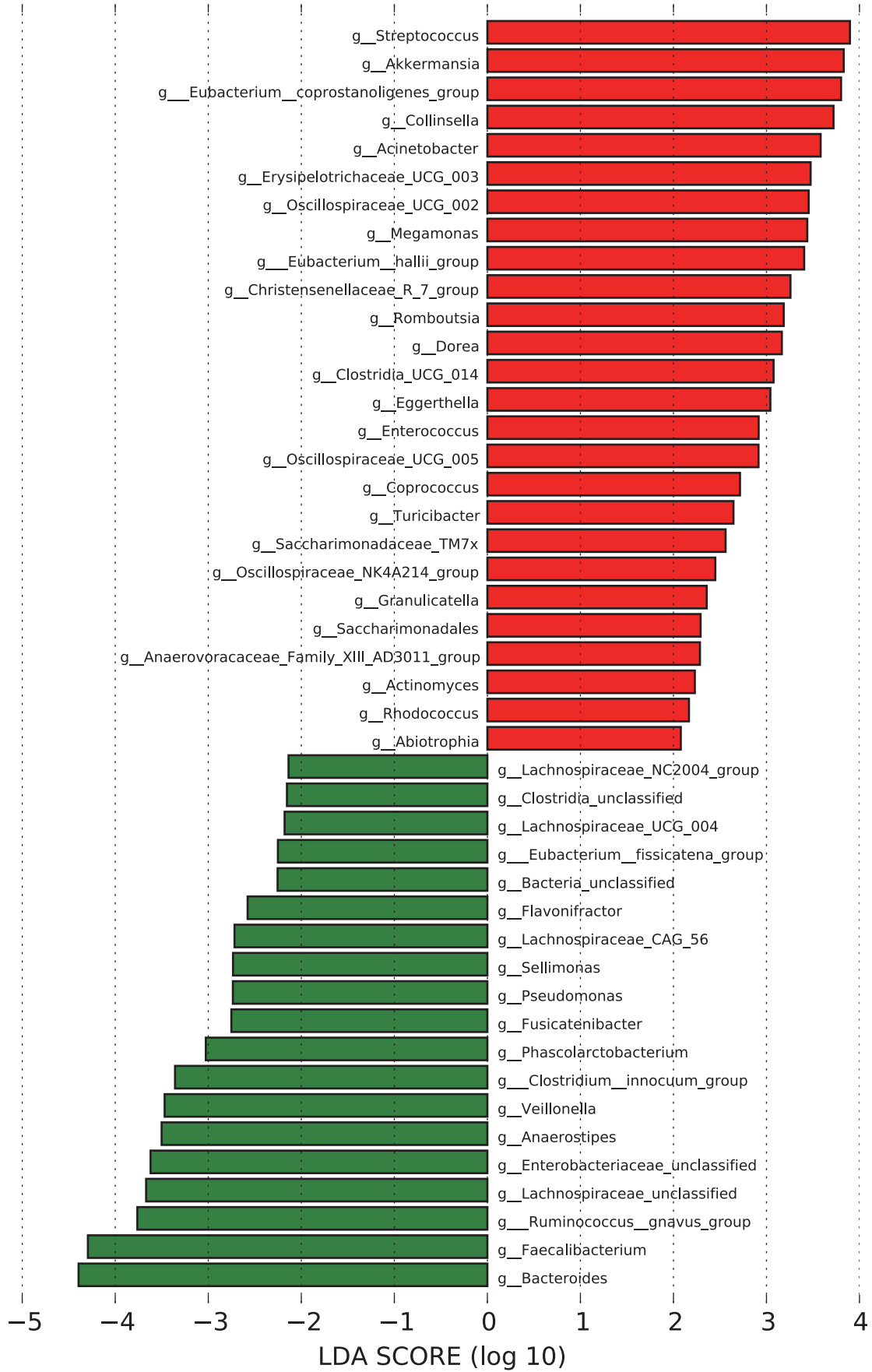

B

AH HC

Cladogram

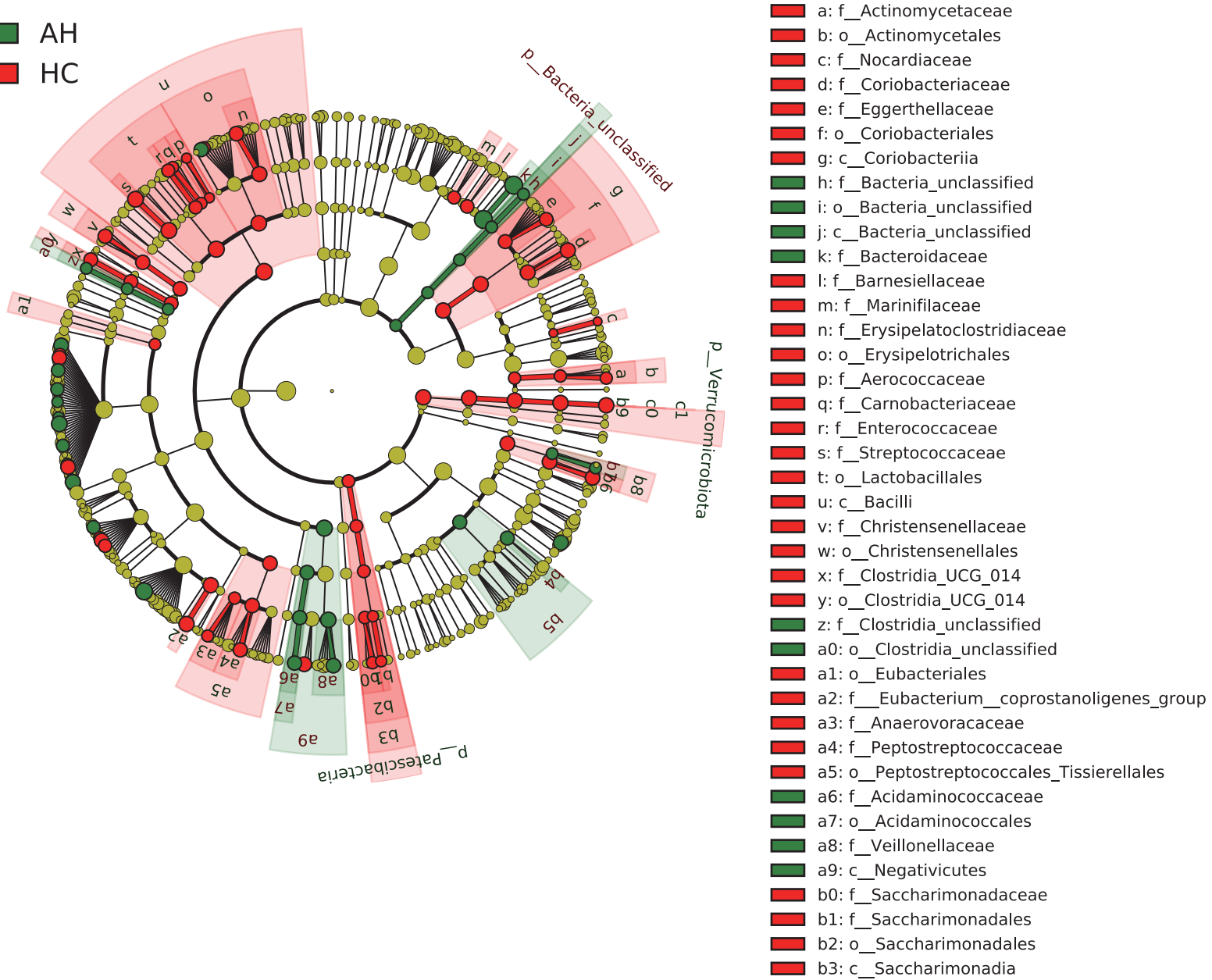

Supplement: Supplementary file 1 [file DataSheet_1.zip › Gut Microbiota Signature in Children with Adenoid Hypertrophy3-hdd-Supplementary Information/Supplementary Figure 6.pdf]

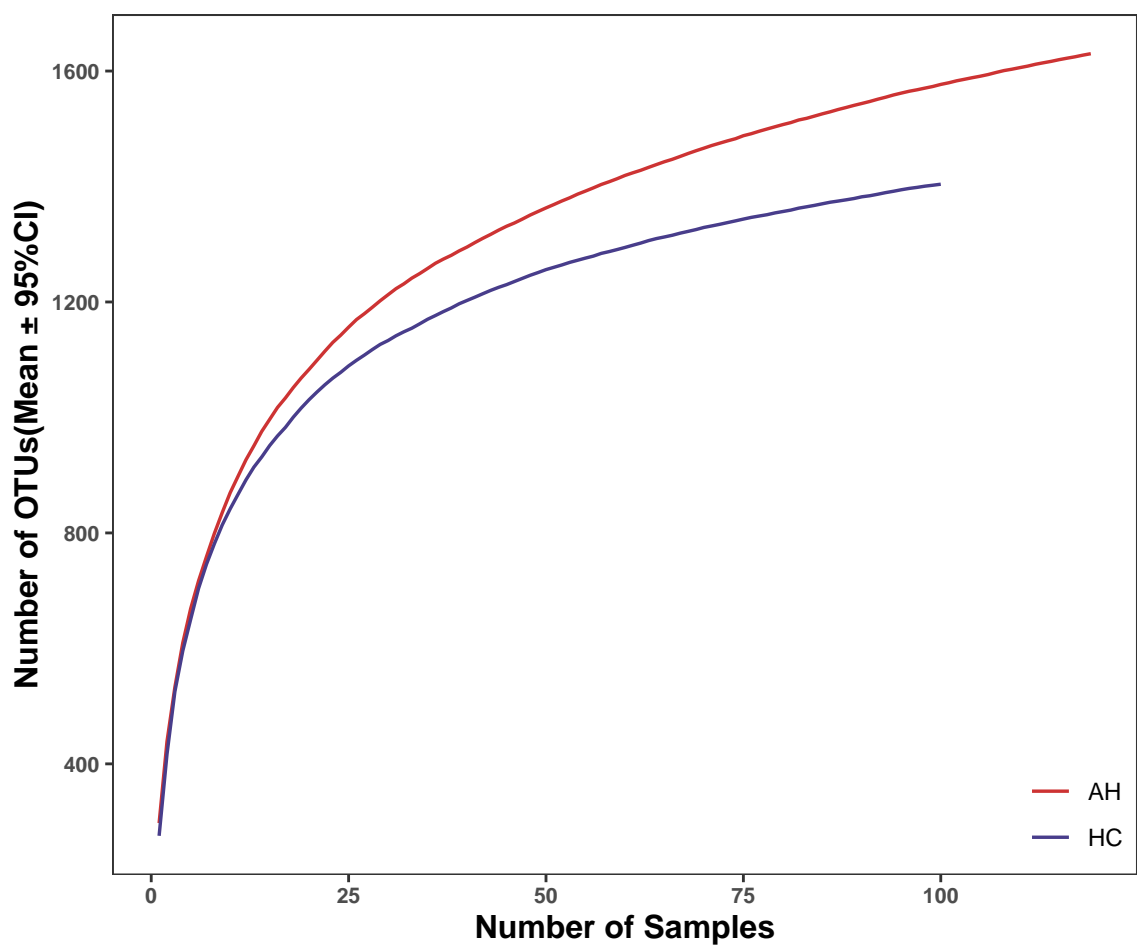

Supplement: Supplementary file 1 [file DataSheet_1.zip › Gut Microbiota Signature in Children with Adenoid Hypertrophy3-hdd-Supplementary Information/Supplementary Figure 2.pdf]

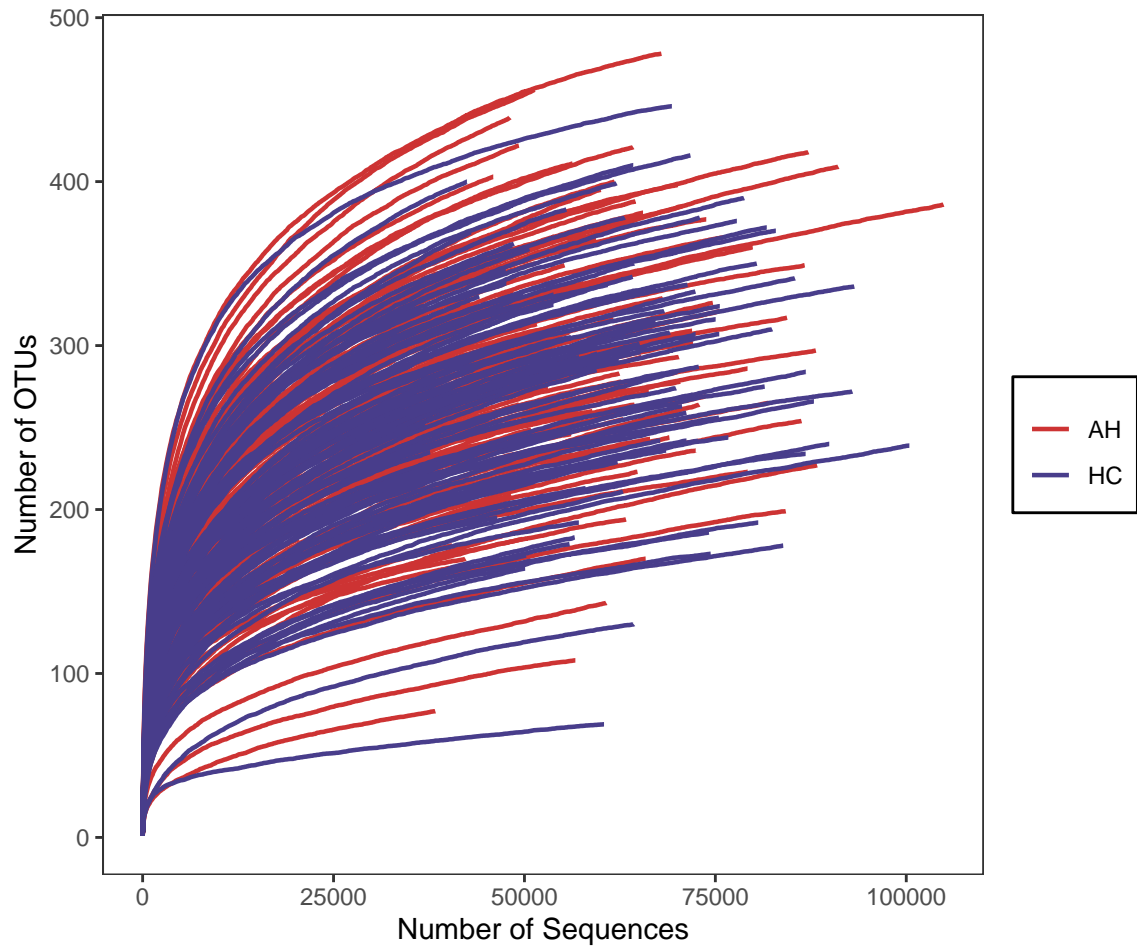

Supplement: Supplementary file 1 [file DataSheet_1.zip › Gut Microbiota Signature in Children with Adenoid Hypertrophy3-hdd-Supplementary Information/Supplementary Figure 3.pdf]

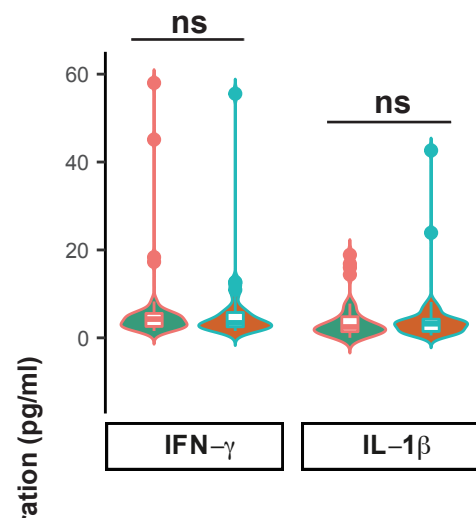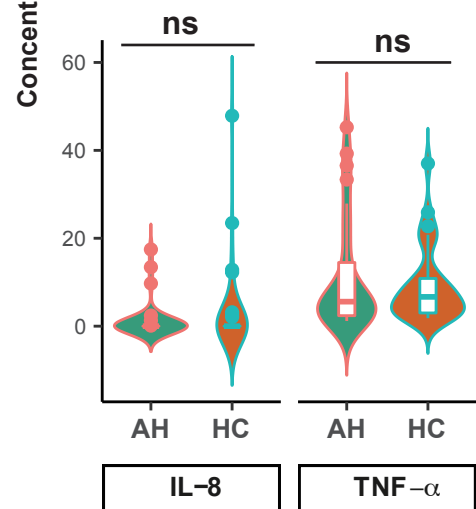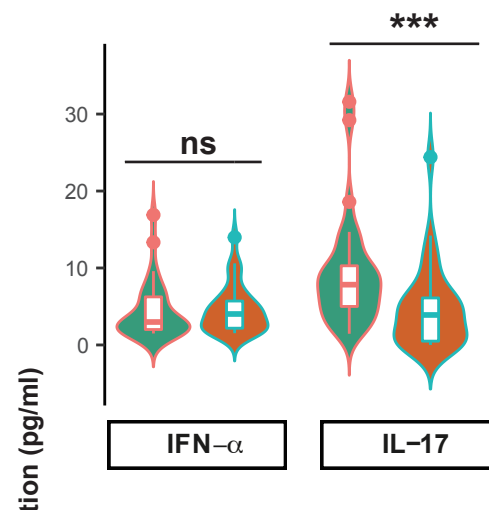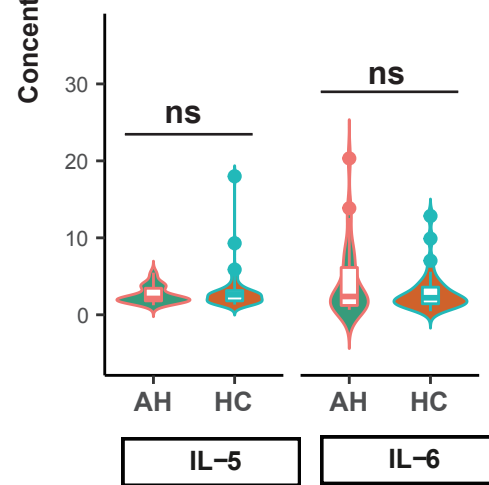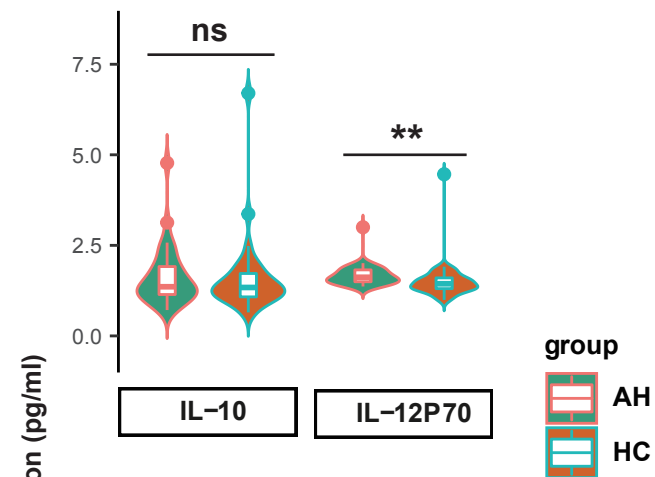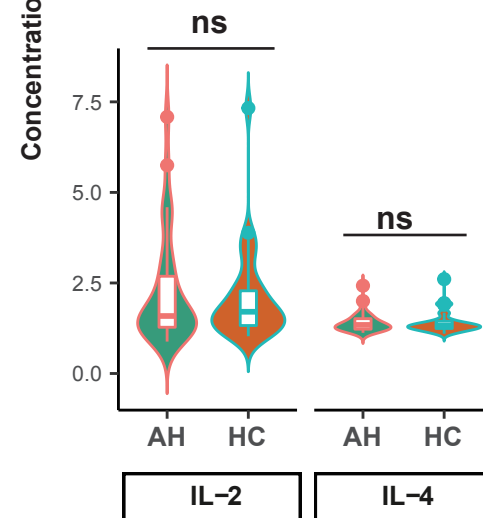

Supplement: Supplementary file 1 [file DataSheet_1.zip › Gut Microbiota Signature in Children with Adenoid Hypertrophy3-hdd-Supplementary Information/Supplementary Figure 1.pdf]

# TLR4

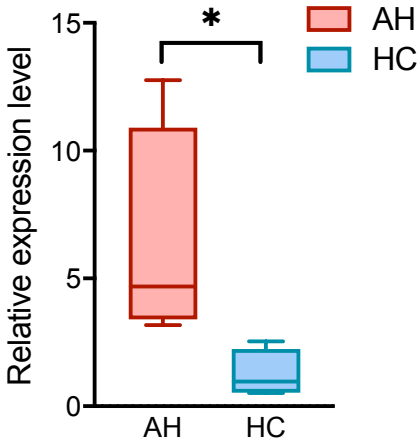

Supplement: Supplementary file 1 [file DataSheet_1.zip › Gut Microbiota Signature in Children with Adenoid Hypertrophy3-hdd-Supplementary Information/Supplementary Figure 10.pdf]
